# Development and validation of screening tool for excessive and problematic use of internet and digital devices (STEPS-IDD) based on the WHO framework (ICD-11) for addictive behaviours

**DOI:** 10.3389/fdgth.2025.1671623

**Published:** 2026-01-06

**Authors:** Yatan Pal Singh Balhara, Swarndeep Singh, Ilika Guha Majumdar, Ayesha Ayoob, Aastha Singh

**Affiliations:** 1 Behavioral Addictions Clinic, National Drug Dependence Treatment Centre (NDDTC), All India Institute of Medical Sciences, New Delhi, India; 2 Vardhman Mahavir Medical College and Safdarjung Hospital, New Delhi, India; 3 Department of Psychology, CHRIST (Deemed to be University), Bengaluru, India

**Keywords:** behavioural addiction, screening, social media use disorder, compulsive buying, problematic pornography use, OTT, gaming, gambling

## Abstract

**Background:**

The widespread use of internet and digital devices has been accompanied by growing concern regarding harms associated with their excessive or problematic use. The World Health Organization has also formally included some of these in its latest classificatory system (ICD-11) under the category of “disorders due to addictive behaviours”. However, a validated, comprehensive screening tool aligned with ICD-11 that screens for these potentially addictive behaviours is lacking. This study aimed to develop and validate the Screening Tool for Excessive and Problematic use of Internet and Digital Devices (STEPS-IDD), designed to assess multiple addictive behaviours based on ICD-11 criteria.

**Methods:**

STEPS-IDD was developed based on the ICD-11 framework for disorders due to addictive behaviours It was applied to assess well-established behavioural addictions like gaming and gambling disorder, as well as less-established but widely researched ones such as problematic use of social media, online shopping/buying, OTT content watching, and pornography watching. Face validity was established through expert review and feedback. Construct validity was evaluated through exploratory factor analysis (EFA), and Cronbach's alpha coefficients were estimated to assess internal consistency. To examine concurrent validity, correlations between scores obtained on the newly developed STEPS-IDD sub-sections and the previously validated Gaming Disorder and Hazardous Gaming Scale (GDHGS) and modified GDHGS for other behaviours were assessed. Receiver Operating Characteristic (ROC) analyses were conducted to determine optimal STEPS-IDD cut-off scores for different behaviours.

**Results:**

Data from a total of 112 college students (64.3% female) with a mean age of 20.5 years were analyzed. STEPS-IDD demonstrated good construct validity, with EFA revealing predominantly unidimensional factor structure for most behavioural domains. Internal consistency was excellent (Cronbach's α = 0.86–0.91 across sub-sections). Concurrent validity was supported by moderate to strong positive correlations (r = 0.44–0.76) of STEPS-IDD sub-sections with corresponding GDHGS and modified GDHGS scores. ROC analyses yielded optimal cut-off scores with high sensitivity and acceptable specificity for different behaviours, and fair to excellent overall diagnostic accuracy.

**Conclusion:**

STEPS-IDD is a psychometrically robust, brief yet comprehensive screening tool grounded in the ICD-11 framework, for the risk stratification in the context of addictive behaviours related to the use of the internet and digital devices.

## Introduction

The rapid advances in internet penetration and the widespread availability of digital devices such as smartphones have been accompanied by a growing concern about the various addictive behaviours associated with their excessive and/or problematic use (1). This includes certain well-recognized conditions such as gaming disorder and gambling disorder (2, 3), as well as several other widely researched, but yet lesser established conditions associated with excessive and problematic use of social media, online shopping/buying, OTT content watching, and pornographic watching, among others (4). These behaviours, when excessive or dysregulated, have been reported to be associated with a range of physical, psychological, social, academic, and occupational harms (5). The younger populations are especially vulnerable and susceptible to experiencing these harms due to their high exposure to digital technology and online platforms and their stage of brain development (6, 7).

In recognition of this growing burden of addictive behaviours, the World Health Organization (WHO) has also recognized “disorders due to addictive behaviours” as a diagnostic category in the latest revision of the International Classification of Diseases (ICD-11). This categorization marks a significant advancement by placing behavioural addictions alongside other addictive disorders such as substance use disorders, thereby acknowledging their clinical relevance, associated distress and dysfunction, and the need for public health attention and further research (8). While, gaming disorder and gambling disorder have been defined with clear essential and additional clinical features; ICD-11 also makes provisions for other, less well-established conditions under the categories of “other specified disorders due to addictive behaviours” and “unspecified disorders due to addictive behaviours”.

Screening tools for addictive disorders have multiple utility (9). To begin with, such tools help identify (and rule out) the presence of a problem. These can also help offer initial insight into the extent of the problem and thereby can categorise the risk levels. While screening tools are not intended to offer a diagnosis, it has been shown that certain risk levels correspond to harmful, hazardous and dependent patterns. Finally, by categorising the risk level, such tools help match the person with the appropriate intervention(s). Such interventions can range from simple advice to more detailed and intensive assessment and interventions. Also, quantification of the risk level helps identify the need for brief intervention. Besides ensuring that appropriate intervention is offered, such tools help ensure the judicious use of health care resources. Alcohol Smoking Substance Involvement Screening Test (ASSIST) and Alcohol Use Disorders Identification Test (AUDIT) are prime examples of such screening tools for addictive disorders that have been widely used across diverse settings and have been found to strengthen the services for such disorders (10, 11). A previous tool for addictive behaviours based on ICD-11 was developed for German speaking population (12).

In the context of addictive behaviours, a major limitation in the current research landscape is the paucity of standardized, validated assessment tools that are aligned with the ICD-11 framework for comprehensively screening people for addictive behaviours associated with the use of internet and digital devices. The available tools are disorder-specific (e.g., focused on gaming or social media), and none of these assess the full range of behaviours that may present with addictive patterns (13–15). In addition, such tools are not linked with the follow-up interventions and hence fail to guide the future course of action based on the findings of the screening. Furthermore, many of the earlier tools have not been updated in accordance with the latest diagnostic features described in the ICD-11, thereby limiting their relevance and clinical utility. As a result, clinicians and researchers face difficulties in systematically identifying and categorizing these behaviours, leading to under-recognition, inconsistent detection, and lack of targeted interventions for their prevention or management.

Thus, the present study aimed to address this critical gap by developing an easy to use, comprehensive, validated screening tool with robust psychometric properties for assessing both well-established as well as other widely researched but as yet less-established addictive behaviours as per the WHO framework described in ICD-11. ICD-11 has introduced the essential and additional characteristics for disorders due to addictive behaviours. In addition to this, we also aimed to determine optimal cut-off scores to categorize the pattern of use into minimal to low- risk, intermediate- risk and high- risk groups; and to examine this new tool's diagnostic performance in terms of sensitivity, specificity, and overall screening accuracy. This shall help in effective screening of the population to identify people at various risk levels for either having or developing various different types of potentially addictive behaviours such as gaming, gambling, problematic use of social media, shopping/buying, OTT content watching, and pornographic watching, among others. The tool shall also be future ready by making provision for inclusion of these currently non-specified addictive behaviours if these are included as specific addictive disorders in the diagnostic systems at a later stage. The tools shall help categorise the risk levels associated with engagement in such behaviours. Finally, such a tool is envisaged to also help in linking the screening findings with the subsequent intervention needed, thereby guiding the course of action following screening in the person.

## Methods

### Study setting and participants

The study was conducted among college students in the northern part of India. Students were eligible if they were 18 years or older, of any gender, could comprehend English, and agreed to provide informed written consent. Students were approached in a classroom setting. They were explained about the purpose and procedures of the study by the research staff. Students who agreed to participate and provide consent were invited to take part in this research.

### Consent and ethical approval declaration

All the participants provided written informed consent prior to enrolment in the study. The study protocol was approved by the Institute Ethics Committee of All India Institute of Medical Sciences, New Delhi prior to the commencement of data collection.

### Instruments

#### Basic socio-demographic proforma

A semi-structured questionnaire designed for collecting basic socio-demographic details such as age, gender and other participant-related characteristics was administered.

#### Gaming disorder and hazardous gaming scale (GDHGS)

This is a validated scale developed to assist trained interviewers in diagnosing gaming disorder and hazardous gaming as per the ICD-11 criteria (15). It consists of five questions rated on a Likert scale (0: Never to 4: Daily or almost daily), and a sixth item assessing impairment across different domains due to the gaming behaviour on a dichotomous (Yes:1/No:0) response format. Total score obtained by summing individual item scores corresponds with the severity or risk of disordered gaming. Spearman correlational analysis showed strong positive correlation of GDGHS score with IGDS-SF score (rs = 0.878, *p* < 0.01). The internal consistency of GDHGS as measured by the Cronbach's alpha was 0.914. Further, the GDHGS did not have its reliability increased by removal of any of the five items included in the scale. Also, the threshold for significant floor and ceiling effect was not reached.

#### Modified GDHGS for other addictive behaviours

This is a modified version of the GDHGS described above. Here, gaming disorder was replaced by other potentially addictive behaviours such as gambling, social media use and others to help trained interviewers identify the extent and severity of different problematic behaviours and associated impairment among study participants. This helped in ensuring a degree of standardization in how all the different potentially addictive behaviours were assessed among the study participants, with higher total scores indicative of greater severity of addictive behaviours. The choice of using this tool was guided by two main factors. First, there is currently a unitary approach to the diagnosis of addictive behaviours in ICD-11. The essential criteria for gaming disorder and gambling disorder (the two specified addictive behaviours in ICD- 11) are exactly the same. Even the additional features of the two are similar with the exception of one feature. Second, there is a lack of a validated and well-established diagnostic tool based on the latest ICD-11 criteria for excessive and problematic shopping/buying, pornography watching, OTT watching, and social media use. Furthermore, the scarce assessment tools that are available, often either capture the behaviours only over the internet medium (excluding or ignoring the digital, but offline engagement), or are based on the older DSM-5 criteria for addictive behaviours such as internet gaming disorder or gambling disorder (16, 17).

#### Generalized problematic internet use scale-2 (GPIUS-2)

This is a validated and well-established questionnaire for assessment of generalized problematic internet use, and has been previously used to measure problematic internet use among college students in India as well as other countries (18). It consists of 15 items, self-rated on a seven-point Likert scale with higher total scores indicating greater severity of problematic internet use. This scale was developed by Caplan based on the cognitive behavioural model of excessive or maladaptive internet use in general, and likely subsumes different types of specific problematic internet use behaviours like playing online games or watching online pornographic content (19).

#### Screening tool for excessive and problematic use of internet and digital devices (STEP- IDD)

This new tool was developed by the study authors to screen the population for excessive and/ or problematic engagement in behaviours which have been broadly described under the rubric of addictive behaviours in the available literature. The items were designed to map both the essential as well as the additional clinical features listed for disorders due to addictive behaviours in the latest International Classification of Diseases (ICD-11). An attempt was made to frame the items in simple language to help lay people or participants easily comprehend and respond to them. The response format used to capture the frequency of particular problem or phenomenon included four mutually exclusive response categories: Never or Rarely (less than 12 times in the last 12 months) = 1; Monthly (1–3 times per month) = 2; Weekly (1–3 times per week) = 3; Daily or Almost Daily (4–7 times per week) = 4. The tool has been developed to be compatible with both self-administration by the participant as well as administration by a lay person in a face-to-face interview format. The first question is a filter question exploring any use of internet or digital devices for engaging in potentially addictive behaviour in the past 12 months (one year). Based on the response to this question (Yes/No format), the remaining ten questions for gambling and nine for other behaviours were asked only for those behaviours that were answered positively in response to the first question. This modular approach allows for introduction of a skip format, and saves administration time required to cover a range of different behaviours such as gaming, gambling, social media use, shopping/buying, OTT content watching, or pornography watching, among others. The final version used for data collection, broadly covered the essential as well as additional clinical characteristics or features of addictive behaviours as listed in the ICD-11 (see Table 1). The score obtained on these ten items (for gambling) and nine items (for other behaviors) were summated to obtain a total score for different potentially addictive behaviours.

**Table 1:** Description of questions comprising the screening tool for excessive and problematic use of internet and digital devices (STEPS-IDD) tool.

Brief description	Item wording
STEPS-IDD Filter question	Q1. In the past 12 months, for which of the following have you used the internet/digital devices?
Item 1 (LOC criteria)	Q2. In the past 12 months, how often have you experienced impaired control (e.g., in terms of its onset, frequency, intensity, duration, termination, or context) over your ________ (specify the behaviour)
Item 2 (Pre-occupation criteria)	Q3. In the past 12 months, how often have you given increasing priority to ________ (specify the behaviour) to the extent that it took precedence over other life interests and daily activities?
Item 3 (Use despite harm criteria)	Q4. In the past 12 months, how often has there been continuation or escalation of_________ (specify the behaviour) despite the occurrence of negative consequences (e.g., family conflict, poor scholastic performance, negative impact on health, marital conflict due to behaviour, repeated and substantial financial losses, etc.)?
Item 4 (Tolerance criteria)	Q5. In the past 12 months, how often have you increased the duration or frequency of _________ (specify the behaviour) over time or experienced a need to engage in _________(specify the behaviour) with increasing levels of complexity or requiring increasing skills or strategy or in a pattern of increasing intensity or increasing amount of money spent in an effort to maintain or exceed previous levels of excitement, or to avoid boredom?
Item 5 (Craving criteria)	Q6. In the past 12 months, how often have you experienced urges or cravings to engage in/ with _________ (specify the behaviour) during other activities?
Item 6 (Withdrawal criteria)	Q7. In the past 12 months, how often have you experienced dysphoria and exhibited adversarial behaviour or verbal or physical aggression upon cessation or reduction of________ (specify the behaviour), whether self-initiated or imposed by others?
Item 7 (Disruption of biological functions criteria)	Q8. In the past 12 months, how often have you experienced substantial disruptions in diet, sleep, exercise and other health-related behaviours that can result in negative physical and mental health outcomes, due to extended periods of ________ (specify the behaviour)?
Item 8 (Deception/Concealment criteria)	Q9. In the past 12 months, how often have you engaged in deceitful behaviour to conceal the extent of your losses from loved ones or attempt to obtain money in order to repay your debts due to________ (specify the behaviour)?
Item 9 (Increased risk of harm criteria)	Q10. In the past 12 months, how often has your ________ (specify the behaviour) led to an increased risk of experiencing harmful physical or mental health consequences to yourself or others?
Item 10 (Distress/Dysfunction criteria)	Q11. In the past 12 months, how often have you experienced marked distress or significant impairment in personal, family, social, educational, occupational, or other important areas of functioning due to ________ (specify the behaviour)?

LOC, loss of control.

### Assessment of study participants

The data for the study were collected from university students enrolled in graduate and postgraduate courses. College students of either gender, aged 18 years or older, able to read and understand English, and willing to provide written informed consent were included in the study. Those who refused consent were excluded from the study. Students were approached in their class. They were explained the study process. Those who expressed interest and offered consent for participation were assessed individually by two trained interviewers. One of the interviewers assessed the participants using the semi- structured questionnaire, and Screening Tool for Excessive and Problematic uSe of Internet and Digital Devices (STEP- IDD). The second interviewer assessed the participants using the Gaming Disorder and Hazardous Gaming Scale (GDHGS), modified Gaming Disorder and Hazardous Gaming Scale (GDHGS) for other addictive behaviors and Generalized Problematic Internet Use Scale-2 (GPIUS-2). The two interviewers conducted their assessment individually and in separate sessions.

### Sample size calculation

According to the commonly accepted guideline, a minimum of 10 participants per item is recommended for the development and validation of a scale measuring a latent construct (20). The STEPS-IDD consisted of a screening question followed by ten-items for assessment of potentially addictive behaviours. Based on this criterion, a minimum sample size of 110 participants was targeted to ensure adequate statistical power for psychometric evaluation.

### Statistical analysis

Statistical analysis was conducted using the Jamovi version 2.3.28 (Jamovi Research, Vienna, Austria) and the SPSS version 26.0 (IBM Corp, Armonk, NY). The data were described using appropriate descriptive statistics. Normality was assessed using the Kolmogorov–Smirnov and Shapiro–Wilk tests, and appropriate parametric or non-parametric inferential statistics were applied according to the results. Six separate exploratory factor analysis (EFA) using the principal axis factoring with direct oblimin rotation method were conducted to examine the overall construct validity of the STEPS-IDD comprising of various sub-sections for different addictive behavioural disorders. Kaiser–Meyer–Olkin's (KMO) measure of sampling adequacy and the Bartlett's test (BT) of sphericity were calculated to assess the suitability of data for EFA. The number of factors to be extracted in the EFA was determined using the Kaiser criterion (eigenvalues greater than one) and visual inspection of the scree plot. Concurrent validity was assessed by conducting the Spearman correlation analyses to examine the relationship between the STEPS-IDD subsection scores and the corresponding GDHGS or modified GDHGS scores for different potentially addictive behaviours. The internal consistency of different subsections of the STEPS-IDD tool was evaluated by calculating the individual Cronbach's alpha coefficients for each subsection. The STEPS-IDD's ability to distinguish participants at high risk of having an addictive behavioural disorder from those at either low or no risk was evaluated by performing Receiver Operating Characteristic (ROC) analyses. To achieve this aim, the STEPS-IDD scores were compared against the standard according to ICD-11 criteria based GDHGS and modified GDHGS items. Youden's index method was used to determine the optimal cut-off points on STEPS-IDD sub-sections. The sensitivity, specificity, positive predictive value, and negative predictive value for these diagnostic thresholds were also tabulated. The diagnostic efficacy of different STEPS-IDD sub-sections was assessed by calculating the area under the ROC curve (AUC). A two-tailed *p*-value of less than 0.05 was considered significant for all tests.

## Results

The study sample comprised a total 112 students, with a mean age of 20.5 years [Range: 18 to 25; Standard deviation (SD): 1.7]. Of these 64.3% (72/112) were female, and one participant chose not to disclose their gender. The response rate was 96%.

In response to the first question evaluating study participants' use of internet or digital devices for various different purposes in the past one year; 77 (68.8%), 11 (9.8%), 112 (100%), 100 (89.3%), 110 (98.2%), and 47 (42.0%) reported “yes” for gaming, gambling, social media use, shopping/buying, OTT content watching, and pornography watching, respectively. Four (4.6%) participants also reported using internet or digital network devices for some other behaviour of interest [investing or tracking stocks (two); website development (one); listening to music or browsing online (one)].

### Face validity of STEPS-IDD

We listed all the items in ICD-11 description of addictive behaviours. These included the relevant content form the description section, essential (required) features, and additional clinical features. The initial draft consisted of twenty-one questions, which were later reduced to eleven questions after initial review and feedback from experts including psychiatrist, addiction psychiatrist, clinical psychologist and public health experts with clinical and research experience in the field of behavioural addictions. The intent was to capture the questions to reflect the ICD- 11 conceptualisation of addictive behaviours. This was done to make the tool concise yet sensitive enough to screen people at risk of developing excessive or problematic use of the internet or digital devices. The first question explicitly asked for use of internet or digital devices for engaging in a range of different potentially addictive behaviours among participants. It was decided to provide a range of different activities such as gaming, gambling, social media use, shopping/buying, OTT content watching, pornography watching, and any other behaviour (to be specified by the respondents) to ensure comprehensive assessment of participants' complete repertoire of engagement on the internet and/ or digital devices (online as well as offline mode). The decision to use a frequency-based response format was made after deliberation among experts, to ensure greater interpretability and standardization of scores obtained for the remaining ten items of the STEPS-IDD tool. Finally, modifications were also made in the wording of a few items to remove any ambiguity in meaning, and improve the ease of understanding for respondents. The final items included in the tool map to various aspects related to addictive behaviours including loss of control; increasing priority; continued or escalated use despite harm; tolerance; craving; withdrawal criteria; disruption of biological functions; deception/concealment (for gambling); increased risk of harm; distress/dysfunction (Table 1).

### Construct validity of STEPS-IDD

Exploratory factor analysis (EFA) was conducted using the principal axis factoring method with oblimin rotation on the items of STEPS-IDD to assess its construct validity. Six separate EFAs were run for each potentially addictive behaviour screened by STEPS-IDD. Results of the KMO measure for sample adequacy and BT of sphericity for suitability of data for EFA were described in the Supplementary File. The KMO coefficient was above the 0.6 cut-off value for all except gaming and gambling sub-sections. Whereas, BT for sphericity reported significant *p*-values of less than 0.05 for all sub-sections indicating suitability of data for EFA. The ideal number of factors to be retained in the EFA was one for all sub-sections except two factor solutions for the gambling sub-section (see Supplementary Figures S1a–f). EFA yielded a predominantly unidimensional underlying structure for the STEPS-IDD tool. Further, the factor loadings obtained using EFA and the total variance explained by the extracted factors have been summarized in Supplementary Tables S1a–f. The individual item factor loadings on the extracted factors were more than 0.4 for all items for all behaviours. This indicates that the scale items are effectively summarizing the underlying factor or construct being measured.

### Reliability of STEPS-IDD

The results of internal consistency assessment for the STEPS-IDD sub-sections were tabulated in Supplementary Tables S2a–f. The Cronbach's alpha values of greater than 0.8 were obtained for all the sub-sections. Also, removal of any one of the items from different sub-sections did not result in any substantial increase in the overall reliability of that STEPS-IDD sub-section.

### Concurrent validity of STEPS-IDD

The concurrent validity of STEPS-IDD was examined by assessing the relationship of different subsection scores with the corresponding total scores obtained on the previously validated GDHGS tool for gaming (based on the ICD-11 criteria for gaming disorder & hazardous gaming) and the modified version of GDHGS for other addictive behaviours. Results of both Pearson and Spearman correlational analyses showed a significant moderate to strong positive correlation for different sub-sections, with correlation coefficients ranging between 0.47 to 0.76 for all of them (see Supplementary Tables S3a–f). This suggests an adequate overall concurrent validity of the STEPS-IDD tool.

### Relationship between GPIUS-2 and STEPS-IDD

Table 2 summarized the results of Spearman correlational analyses conducted to explore the relationship between generalized problematic internet use measured using the GPIUS-2 scale score and severity of different potentially addictive behaviours assessed through different subsections of the STEPS-IDD tool. There was a significant positive correlation observed between the STEPS-IDD subsection scores for social media use, shopping/buying, and OTT content watching, and the total GPIUS-2 score indicative of generalized problematic internet use. However, the coefficient was weak for shopping/buying, and OTT content watching and only moderate for shopping/buying. There was no significant correlation of the GPIUS-2 score with gaming, gambling, or pornography watching subsection scores.

**Table 2:** Results of spearman correlational analyses between generalized problematic internet Use scale (GPIUS-2) and screening tool for excessive and problematic use of internet and digital devices (STEPS-IDD) scale scores.

Statistical test		Gaming-STEPS-IDD sub-section score	Gambling-STEPS-IDD sub-section score^#^	SMU-STEPS-IDD sub-section score	Shopping/Buying-STEPS-IDD sub-section score	OTT content watching-STEPS-IDD sub-section score	Pornography watching-STEPS-IDD sub-section score
GPIUS-2 Total score	Spearman's rho (r_s_)	0.106	0.132	0.637*	0.359*	0.342*	0.157
	Degreed of freedom (df)	110	110	110	110	110	110
	*p*-value	0.266	0.164	<0.001	<0.001	<0.001	0.099
	Total (N)	112	112	112	112	112	112

STEPS-IDD, Screening tool for excessive and problematic use of internet and digital devices; GPIUS-2, generalized problematic internet use scale.

* *p*-value < 0.05.

# Item 8 was included only in gambling sub-section score.

### Analysis of STEPS-IDD score distribution: floor and ceiling effects

The frequency distribution of total sub-section wise STEPS-IDD scores corresponding to six different potentially addictive behaviours were described in Supplementary Tables S4a–f. Since participants who screened negative for a particular behaviour initially, would have a score of zero; we used the total sub-section score of one as the lowest value for assessing the floor effects. Similarly, the maximum total score of 30 (for gambling) and 27 (for other behaviours) that was possible for any of the STEPS-IDD sub-section, was used for assessing the ceiling effects. According to the available literature, a significant ceiling or floor effect is considered to be present when 15% or more study participants achieve the maximum or minimum possible score on the scale (21). A total of 11 (9.8%), 1 (0.9%), 11 (9.8%), 6 (5.4%), and 12 (10.7%) participants reported total sub-section score of 10 (i.e., minimum possible value for those reporting positively to the screening question) for gaming, social media use, shopping/buying, OTT content watching, and pornography watching sub-sections, respectively and 3 (2.7%) for sub-section score of 11 for gambling. Whereas the highest score observed was less than 40 (for gambling) or 36 (for other behaviors) (i.e., maximum possible score) for all the subsections of STEPS-IDD. Thus, the threshold for significant ceiling or floor effects was not breached for the STEPS-IDD tool in the study sample.

### Proposed thresholds for classifying participants into high, low, and no risk categories using the STEPS-IDD scores

The previously validated GDHGS instrument and modified GDHGS versions were used to assess the diagnostic criteria met for disordered gaming and other addictive behaviours, respectively in this study. The participants were dichotomised based on score of a 3 or 4 on at least one of the five items on the GDHGS or modified GDHGS. A total of 9 (8.0%), 3 (2.7%), 50 (44.6%), 7 (6.3%), 33 (29.5%), and 6 (5.4%) participants were organised in one group and the rest to the other for gaming, gambling, social media use, shopping or buying, OTT content watching, and pornography watching separately. Six separate Receiver Operating Characteristic (ROC) curves were constructed to determine the respective cut-off scores for gaming, gambling, social media use, shopping or buying, OTT content watching, and pornography watching on the STEPS-IDD (see Supplementary Figures S2a–f). The area under the curve for gaming, gambling, social media use, shopping/ buying, OTT content watching, and pornography watching was 0.929 (95% CI: 0.880–0.978), 1.00 (95% CI: 1.00–1.00), 0.773 (95% CI: 0.684–0.862), 0.902 (95% CI: 0.802–1.000), 0.754 (95% CI: 0.656–0.851), and 0.839 (95% CI: 0.645–1.000), respectively. The sensitivity, specificity, Youden's index, positive predictive value, and negative predictive values for different sub-sections of the STEPS-IDD tool have been described in Supplementary Tables S5a–f.

Based on the Youden's index method for determining optimal cut-off scores; the thresholds obtained were 16 (100% sensitivity & 86.4% specificity), 21 (100% sensitivity & 100% specificity), 24 (58.0% sensitivity & 88.7% specificity), 13 (100% sensitivity & 66.6% specificity), 17 (93.9% sensitivity & 46.8% specificity), and 10 (83.3% sensitivity & 74.5% specificity) for identifying participants at high risk of having excessive and/or problematic (including related addictive behaviour disorders) gaming, gambling, social media use, shopping or buying, OTT content watching, and pornography watching, respectively. Of the rest of the participants who obtained a score of ten (for gambling) and nine (for other behaviours) on STEPS-IDD in the past 12 months (one year), they were considered to be at minimal to low risk. Whereas the remaining participants were classified to be at intermediate risk (Table 3).

**Table 3:** Determination of risk levels for different addictive behaviours according to the tentative STEPS-IDD sub-section cut-off scores.

Behavior	Minimal to low risk	Intermediate risk	High risk
a. Gaming	9	10–15	16 or more
b. Gambling	10	11–20	21 or more
c. Social media use	9	10–23	24 or more
d. Shopping/ buying	9	10–12	13 or more
e. OTT content watching	9	10–16	17 or more
f. Pornography watching	9	–	10 or more
g. Any other behaviour(s) of interest (specify)___________	9	–	10 or more

STEPS-IDD, Screening tool for excessive and problematic use of internet and digital devices.

## Discussion

This study describes the development and validation of a brief yet comprehensive screening tool, namely the Screening Tool for Excessive and Problematic uSe of Internet and Digital Devices (STEPS-IDD) based on the ICD-11 diagnostic framework for disorders due to addictive behaviours. The need to strengthen research on addictive behaviors in India has been identified earlier (22). Our findings demonstrated robust psychometric properties of the STEPS-IDD tool, including acceptable construct and concurrent validity, along with excellent internal consistency. Its diagnostic performance in distinguishing individuals at high risk of addictive behavior from those at low or no risk, across different domains such as gaming, gambling, social media use, shopping/buying, OTT content watching, and pornography watching was found to be satisfactory.

The development of STEPS-IDD was grounded in the diagnostic framework outlined in the latest ICD-11 criteria for disorders due to addictive behaviours, and involved item refinement in consultation with mental health, addiction and public health experts to enhance its face validity. It integrated both essential and additional clinical features listed in the ICD-11 into a single, adaptable tool that can be used for assessing potentially addictive behaviours related to internet and digital device use across different behavioural domains. An 11-item tool was finalized to screen for both well-established addictive behaviour disorders such as gaming and gambling, as well as widely researched, yet less-established potentially addictive behaviours such as social media use, shopping/buying, OTT content watching, pornography watching, and any other specific behaviour.

The construct validity of the STEPS-IDD was supported by the results of exploratory factor analyses (EFA), with a single factor solution obtained for all sub-sections except for gambling which yielded a two-factor solution. This suggested a predominantly unidimensional structure, consistent with the theoretical model of a singular underlying addictive process or latent construct assessed by the STEPS-IDD items. This was also in line with the previous research finding, that reported unidimensional structure for other screening or diagnostic instruments that were developed based on the ICD-11 diagnostic framework for potentially addictive behaviours such as gaming (15, 23). Furthermore, all items demonstrated factor loadings above the generally accepted threshold of 0.40 (24), indicating that they contribute meaningfully to the constructs being measured.

The internal consistency of STEPS-IDD was excellent, with Cronbach's alpha values ranging between 0.8–0.9 for different sub-sections (25). Also, there was no substantial change in the Cronbach's alpha value upon deletion of any single item from the sub-section. This suggests that the items within each sub-section were reliably measuring the same underlying construct, and that no single item was unduly distorting the overall consistency.

We also explored the relationship between different potentially addictive behaviours involving use of internet and digital devices (STEPS-IDD subsection scores) and generalized problematic internet use (GPIUS-2 score). Importantly, there was at best a moderate positive correlation of GPIUS-2 score with STEPS-IDD sub-section score for social media use; while a weak positive correlation was observed for shopping/buying and OTT content watching sub-sections. Moreover, no significant correlation was found between GPIUS-2 and STEPS-IDD scores for gaming, gambling, and pornography watching. This possibly suggests that problematic engagement in these behaviours might follow a more distinct pattern rather than being a part of the generalized internet overuse problem. This nuanced relationship observed with the GPIUS-2 score, suggests that the STEPS-IDD instrument measures a distinct construct from the generalized problematic internet use. While the debate on generalised or specific internet use related addictions is ongoing in the literature our previous research suggested that internet gaming disorder and generalized problematic internet use are conceptually distinct entities (26).

Concurrent validity was examined by evaluating the correlation of different STEPS-IDD subsection scores with those obtained from the previously validated assessment tool (i.e., GDHGS) based on the ICD-11 core criteria for disordered gaming, and its modified versions which served as the reference standard in the absence of other validated ICD-11–aligned tools for addictive behaviours other than gaming. The correlation coefficients ranged between 0.47 and 0.76 for different STEPS-IDD subsection scores, indicating a moderate to strong positive correlation. These findings support the conceptual alignment of STEPS-IDD with other existing instruments designed to measure addictive behaviours based on the ICD-11 core criteria of gaming disorder and hazardous gaming.

The diagnostic performance of the STEPS-IDD tool in distinguishing high-risk participants from the rest of the sample evaluated through ROC analysis was promising. The AUC values for different sub-sections ranged between 0.75 and 1.00, indicating fair to excellent discriminatory power of the STEPS-IDD tool (27). This ensures that those at high risk of addictive behaviours are not missed during the screening process. Youden's index method was used to derive optimal cut-off points balancing both sensitivity and specificity for different subsections of STEPS-IDD. The Youden's index method helped identify cut-off points that provided the best balance between sensitivity and specificity; and ensured that the proposed threshold scores maximized the correct identification of true positives (i.e., sensitivity) while minimizing the false positive rates (i.e., 1-specificity). This is particularly useful in clinical or screening contexts where both missing actual cases (false negatives) and misidentification of non-cases (false positives) can have important consequences. In this study, by using Youden's index we determined cut-off scores that would optimize diagnostic accuracy for detecting high-risk individuals across different potentially addictive behaviours. The tentative cut-off score of 16 and 21 for the well-established addictive behaviours in form of gaming and gambling, respectively yielded 100% sensitivity for both these behaviours in the study sample, but specificity differed (86.4% vs. 100%; suggesting that gambling-related problematic engagement may be more accurately differentiated from non-pathological engagement patterns by the STEPS-IDD items. While the tentative cut-off score of 17 for OTT content watching in this study reported a high level of sensitivity (93.9%) in identifying individuals at high-risk, it failed to achieve a good degree of specificity (46.8%); suggesting it to be acceptable only for initial screening of large population samples. Being a screening tool we found this trade-off between the sensitivity and specificity to be acceptable. A much higher tentative cut-off score of 24 for social media use resulting in modest levels of sensitivity and specificity in identifying individuals at high-risk; might be reflective of the socially normative use of social media among the young adult population, and only high degree of engagement or symptoms associated with high-risk of social media use disorder. On the other hand, a much lower tentative cut-off score of 10 was noted for pornography watching (yielding 83.3% sensitivity), respectively. This suggests that a lesser degree of symptom or STEPS-IDD item endorsement could be sufficient to signal high-risk of problematic patterns of this behaviour. Another possible explanation for this low cut-off score observed for the pornography watching could be due to social desirability bias and underreporting by participants. Unlike other behavioural domains assessed (e.g., gaming, social media use), pornography use tends to carry a greater degree of social stigma and cultural taboo, especially in more conservative or collectivist societies like India (28). As a result, individuals might be less willing to disclose the true extent or frequency of their engagement with pornography. Nonetheless, the wide variation noted in optimal cut-off scores for identifying high-risk individuals across different behaviours involving use of internet and digital devices might reflect the inherent differences in the characteristics and underlying purpose or motivations behind engagement in these different potentially addictive behaviours. A “one-size-fits-all” threshold could misclassify individuals by either missing true cases or wrongly labelling non-problematic users. These findings support the need for having tailored threshold scores or criteria for different addictive behaviours. Tentative cut-off scores have been proposed for screening at high-risk individuals for different addictive behaviours. Notably, a sensitivity of 100% was achieved with the proposed cut-off scores for gaming and gambling highlighting the potential utility of STEPS-IDD for population-level screening and early intervention.

Furthermore, none of the gaming, gambling, social media use, shopping/buying, OTT content watching, pornography watching demonstrated significant floor or ceiling effects, with the percentage of participants with minimum and maximum possible scores being well below the typical 15% threshold (21). The absence of significant floor or ceiling effects in the STEPS-IDD score distributions further supports the appropriateness of the tool for screening across varying levels of severity, without the risk of scores clustering at the extremes. This suggests that the tool can sensitively differentiate between individuals with varying degrees of severity and problematic engagement.

To the best of our knowledge, this is the first validated brief yet comprehensive English language screening tool that fully incorporates both the essential and additional ICD-11 diagnostic features for disorders due to addictive behaviours, while simultaneously assessing individuals for multiple different types of excessive and problematic addictive behaviours (online and offline) related to the use of internet and digital devices. A previous tool [Assessment of Criteria for Specific Internet-use Disorders (ACSID-11)] based on ICD-11 was developed for German speaking population (12). However, it only included items from the essential criteria for addictive behaviours as listed in ICD-11. Also, this tool included only gaming, online shopping, use of online pornography, use of social-networks, and online gambling. In addition, the tool deviated from the ICD- 11 conceptualization of addictive behaviours as it assessed for intensity besides the frequency of engagement of behaviour. The ICD- 11 criteria does not assess the level of intensity of engagement in the behaviour as a diagnostic requirement. The STEPS-IDD incorporated not only the essential but also the additional clinical features of behavioural addictions. In addition, it screened for both online and offline behaviours involving use of digital devices. The STEPS- IDD also operationalises the frequency of engagement in specific behaviours (for example, Never or Rarely refers to less than 12 times in last 12 months). Finally, STEPS-IDD included an item to capture the hazardous pattern of engaging in addictive behaviour. This is often overlooked in the screening tools for addictive behaviours.

Another important strength of the STEPS-IDD is its practical design. The use of a modular format with a skip pattern or logic, helps reduce respondent burden while allowing for assessment of multiple different potentially addictive behaviours. Furthermore, the tool is designed to be usable in both self-report and interviewer-administered formats, enhancing its feasibility for use in research as well as clinical settings. Thus the tool may be used for self-evaluation. The simple framing of the items on the tool makes it easy to use, precluding the requirement for specialised qualifications and intensive training for its use. The tool can be used in diverse settings including various levels (primary, secondary, tertiary) of health care settings, educational settings, workplace settings, community settings, among others.

STEPS- IDD also fills another major lacunae of the existing screening tools. While the existing screening tools offer an output in terms of score, they fail to offer any guidance on the subsequent course of action. By offering stratification of the risk levels the tool also offers an opportunity to match the intervention to the risk levels. Those with minimal to no risk could be offered information on the potentially addictive behaviours and the consequences associated with excessive and problematic engagement in these behaviours, along with professional advice to engage in these behaviours mindfully. Persons with intermediate-risk will require brief intervention targeted at modification of behaviours with an aim to mitigate or reduce the risk. Finally, those at high-risk would benefit from brief intervention followed by referral for more detailed assessment and management.

Despite these strengths, there are certain limitations that must be acknowledged. First, the study sample consisted exclusively of college students from the northern part of India, which may limit the generalizability of results to other populations or age groups. The questionnaire was administered by the interviewer. The future studies should asses the questionnaire in more diverse population groups including clinical settings. The KMO values for gaming and gambling sub-sections of STEPS-IDD were less than 0.6, suggesting need for larger sample size for conducting EFA for these domains. It is proposed to do a follow-up study among a diverse group of students from different parts of the country. Second, the reference standards used for comparing the STEPS-IDD performance via ROC analysis in this study might not be an ideal method. Although GDHGS and its modified versions were aligned with the ICD-11 core criteria for behavioural addictions, they are still in the early stages of adoption and lack widespread use and validation for potentially addictive behaviours other than gaming. A lack of an alternative ICD-11 based tool for these potentially addictive behaviours precluded any alternative approach. We propose that the use of structured clinical interview by a trained mental health professional for establishing the diagnosis and severity of addictive behaviour disorder as per the ICD-11 criteria in future studies shall help further establish the criterion validity of STEPS-IDD in subsequent studies. Third, test-retest reliability and predictive validity of the STEPS-IDD were not assessed in this study and should be explored in future research. Finally, while the STEPS-IDD makes provisions for screening for other addictive behaviours that have not been specified by name (viz. gaming, gambling, social media use, shopping/buying, OTT content watching, pornography watching) the sample size for these additional behaviours was not adequate to determine the cut off scores for different risk categories for them.

## Conclusion

The STEPS-IDD is a novel, brief yet comprehensive screening tool grounded in the ICD-11 framework for the early identification of excessive and problematic behaviours (including addictive disorders) related to the use the internet and digital devices. This tool demonstrated robust psychometric properties, with good diagnostic performance in identifying individuals at high risk of having addictive behaviours beyond the traditionally recognized gaming and gambling disorders. Future research is needed to validate its use in diverse populations and to assess its utility in guiding early detection and treatment as well as implementation of preventive interventions over time.

## Data Availability

The raw data supporting the conclusions of this article will be made available by the authors, upon reasonable request to the corresponding author.
