# Supplementary material for: Development and validation of screening tool for excessive and problematic use of internet and digital devices (STEPS-IDD) based on the WHO framework (ICD-11) for addictive behaviours
Source: Front Digit Health. 2026 Jan 6;7:1671623. doi: 10.3389/fdgth.2025.1671623 (PMC12816239; doi:10.3389/fdgth.2025.1671623)

**Results of Exploratory Factor Analysis for Gaming sub-section of the STEPS-IDD tool:**

Kaiser-Meyer-Olkin (KMO) coefficient= 0.50

Bartlett's Test (BT) of sphericity revealed significant p-value (<0.001)

**Supplementary Table S-1a: Summary of results obtained from exploratory factor analysis (EFA) on nine items of the STEPS-IDD sub-section for gaming**

| **Items^#^** | **Factor Loadings ^a,b^** | | **Uniqueness** |
| --- | --- | --- | --- |
|  | **Factor 1** | **Factor 2** |  |
| 1 | 0.672 | - | 0.548 |
| 2 | 0.779 | - | 0.394 |
| 3 | 0.809 | - | 0.346 |
| 4 | 0.824 | - | 0.322 |
| 5 | 0.727 | - | 0.472 |
| 6 | 0.830 | - | 0.312 |
| 7 | 0.830 | - | 0.312 |
| 9 | 0.546 | - | 0.702 |
| 10 | 0.617 | - | 0.619 |
|  | ^a^ Percentage of the Total Variance Explained = 55.3%. ^b^ Only one factor was possible to be extracted from the EFA.  ^#^ Item 8 is included only for gambling sub-section of STEPS-IDD based on the ICD-11 criteria. | | |

**Supplementary Figure S-1a: Scree Plot for exploratory factor analysis (EFA) of the STEPS-IDD sub-section items for gaming**

### Scree Plot


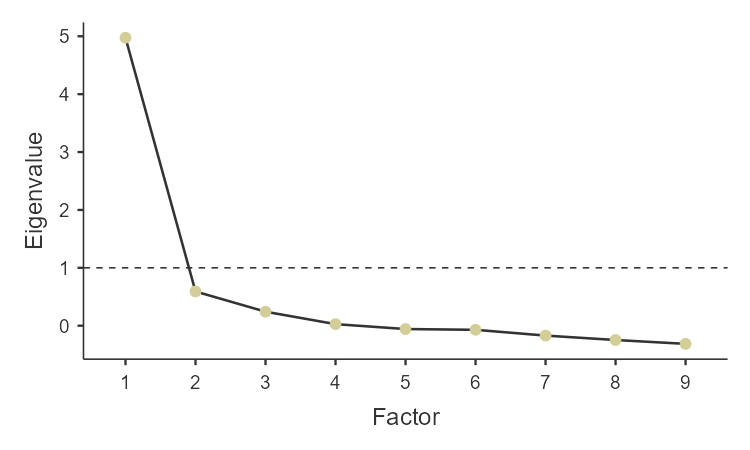


| **Supplementary Table S-2a: Internal consistency for nine items in the gaming sub-section of STEPS-IDD tool** | | |
| --- | --- | --- |
|  | **Cronbach's Alpha value** | **Cronbach's Alpha if item deleted** |
| Complete scale | 0.912 | N/A |
| Item 1 | N/A | 0.907 |
| Item 2 | N/A | 0.898 |
| Item 3 | N/A | 0.896 |
| Item 4 | N/A | 0.896 |
| Item 5 | N/A | 0.903 |
| Item 6 | N/A | 0.898 |
| Item 7 | N/A | 0.898 |
| Item 9 | N/A | 0.913 |
| Item 10 | N/A | 0.909 |
| N/A: Not applicable | | |

**Supplementary Table S-3a: Results of Pearson and Spearman correlational analyses between the scores obtained on the STEPS-IDD gaming sub-section and the GDHGS for gaming**

| Correlation Matrix | | | | | | | |
| --- | --- | --- | --- | --- | --- | --- | --- |
|  | |  | | **Gaming_STEPS_Total** | | **GDHGS_Total** | |
| **Gaming_STEPS_Total** |  | Pearson's r |  | — |  |  |  |
|  |  | df |  | — |  |  |  |
|  |  | p-value |  | — |  |  |  |
|  |  | Spearman's rho |  | — |  |  |  |
|  |  | df |  | — |  |  |  |
|  |  | p-value |  | — |  |  |  |
| **GDHGS_Total** |  | Pearson's r |  | **0.556** | *** | — |  |
|  |  | df |  | 110 |  | — |  |
|  |  | p-value |  | < 0.001 |  | — |  |
|  |  | Spearman's rho |  | **0.714** | *** | — |  |
|  |  | df |  | 110 |  | — |  |
|  |  | p-value |  | < 0.001 |  | — |  |
| Note. * p < .05, ** p < .01, *** p < .001 | | | | | | | |
|  | | | | | | | |

**Supplementary Table S-4a: Frequency distribution table for total score on gaming sub-section of STEPS-IDD**

| **Gaming_STEPS_Total** | | | | | | |
| --- | --- | --- | --- | --- | --- | --- |
|  | | **Frequency** | **Percent** | **Valid Percent** | **Cumulative Percent** |  |
| Valid | 9 | 56 | 50.0 | 50.0 | 50.0 |  |
|  | 10 | 11 | 9.8 | 9.8 | 59.8 |  |
|  | 11 | 3 | 2.7 | 2.7 | 62.5 |  |
|  | 12 | 5 | 4.5 | 4.5 | 67.0 |  |
|  | 13 | 4 | 3.6 | 3.6 | 70.5 |  |
|  | 14 | 5 | 4.5 | 4.5 | 75.0 |  |
|  | 15 | 5 | 4.5 | 4.5 | 79.5 |  |
|  | 16 | 4 | 3.6 | 3.6 | 83.0 |  |
|  | 17 | 6 | 5.4 | 5.4 | 88.4 |  |
|  | 18 | 1 | .9 | .9 | 89.3 |  |
|  | 20 | 4 | 3.6 | 3.6 | 92.9 |  |
|  | 22 | 2 | 1.8 | 1.8 | 94.6 |  |
|  | 24 | 2 | 1.8 | 1.8 | 96.4 |  |
|  | 26 | 2 | 1.8 | 1.8 | 98.2 |  |
|  | 28 | 1 | .9 | .9 | 99.1 |  |
|  | 33 | 1 | .9 | .9 | 100.0 |  |
|  | Total | 112 | 100.0 | 100.0 |  |  |

**Supplementary Table S-5a: Summary output of the Receiver Operating Characteristic (ROC) analysis for the gaming sub-section of STEPS-IDD**

| Scale: Gaming_STEPS_Total | | | | | | | | | | | | | | | |
| --- | --- | --- | --- | --- | --- | --- | --- | --- | --- | --- | --- | --- | --- | --- | --- |
| **Cutpoint** | | **Sensitivity (%)** | | **Specificity (%)** | | **PPV (%)** | | **NPV (%)** | | **Youden's index** | | **AUC** | | **Metric Score** | |
| 9 |  | 100% |  | 0% |  | 8.04% |  | NaN% |  | 0.00000 |  | 0.929 |  | 0.0804 |  |
| 10 |  | 100% |  | 54.37% |  | 16.07% |  | 100% |  | 0.54369 |  | 0.929 |  | 0.5804 |  |
| 11 |  | 100% |  | 65.05% |  | 20% |  | 100% |  | 0.65049 |  | 0.929 |  | 0.6786 |  |
| 12 |  | 100% |  | 67.96% |  | 21.43% |  | 100% |  | 0.67961 |  | 0.929 |  | 0.7054 |  |
| 13 |  | 100% |  | 72.82% |  | 24.32% |  | 100% |  | 0.72816 |  | 0.929 |  | 0.7500 |  |
| 14 |  | 100% |  | 76.7% |  | 27.27% |  | 100% |  | 0.76699 |  | 0.929 |  | 0.7857 |  |
| 15 |  | 100% |  | 81.55% |  | 32.14% |  | 100% |  | 0.81553 |  | 0.929 |  | 0.8304 |  |
| 16 |  | 100% |  | 86.41% |  | 39.13% |  | 100% |  | 0.86408 |  | 0.929 |  | 0.8750 |  |
| 17 |  | 88.89% |  | 89.32% |  | 42.11% |  | 98.92% |  | 0.78209 |  | 0.929 |  | 0.8929 |  |
| 18 |  | 44.44% |  | 91.26% |  | 30.77% |  | 94.95% |  | 0.35707 |  | 0.929 |  | 0.8750 |  |
| 20 |  | 44.44% |  | 92.23% |  | 33.33% |  | 95% |  | 0.36677 |  | 0.929 |  | 0.8839 |  |
| 22 |  | 33.33% |  | 95.15% |  | 37.5% |  | 94.23% |  | 0.28479 |  | 0.929 |  | 0.9018 |  |
| 24 |  | 33.33% |  | 97.09% |  | 50% |  | 94.34% |  | 0.30421 |  | 0.929 |  | 0.9196 |  |
| 26 |  | 11.11% |  | 97.09% |  | 25% |  | 92.59% |  | 0.08198 |  | 0.929 |  | 0.9018 |  |
| 28 |  | 11.11% |  | 99.03% |  | 50% |  | 92.73% |  | 0.10140 |  | 0.929 |  | 0.9196 |  |
| 33 |  | 0% |  | 99.03% |  | 0% |  | 91.89% |  | -0.00971 |  | 0.929 |  | 0.9107 |  |
|  | | | | | | | | | | | | | | | |

**Supplementary Figure S-2a: Receiver Operating Characteristic (ROC) curve for the gaming sub-section of STEPS-IDD**


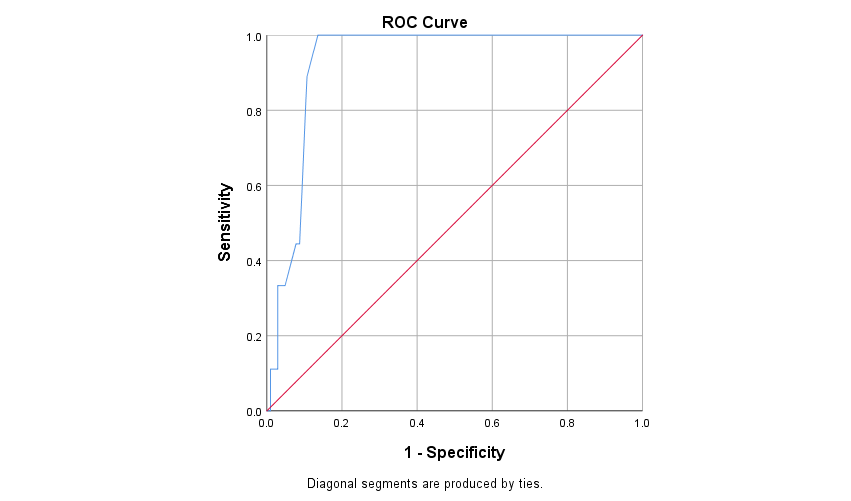


**Results of Exploratory Factor Analysis for Gambling sub-section of the STEPS-IDD tool:**

Kaiser-Meyer-Olkin (KMO) coefficient= 0.56

Bartlett's Test (BT) of sphericity revealed significant p-value (<0.001)

**Supplementary Table S-1b: Summary of results obtained from exploratory factor analysis (EFA) on ten items of the STEPS-IDD sub-section for gambling**

| **Item** | **Factor Loadings ^a,b^** | | **Uniqueness** |
| --- | --- | --- | --- |
|  | **Factor 1** | **Factor 2** |  |
| 1 |  | 0.506 | 0.487 |
| 2 |  | 1.052 | -0.108 |
| 3 |  | 0.730 | 0.474 |
| 4 | 0.725 |  | 0.440 |
| 5 | 0.901 |  | 0.155 |
| 6 | 0.829 |  | 0.352 |
| 7 | 0.452 |  | 0.808 |
| 8 | 0.912 |  | 0.154 |
| 9 | 0.740 |  | 0.210 |
| 10 | 0.583 |  | 0.684 |
|  | ^a^ Percentage of the Total Variance Explained = 63.4%. ^b^ Two factors with eigenvalues >1 were extracted from the EFA. | | |

**Supplementary Figure S1-b: Scree Plot for exploratory factor analysis (EFA) of the STEPS-IDD sub-section items for gambling**


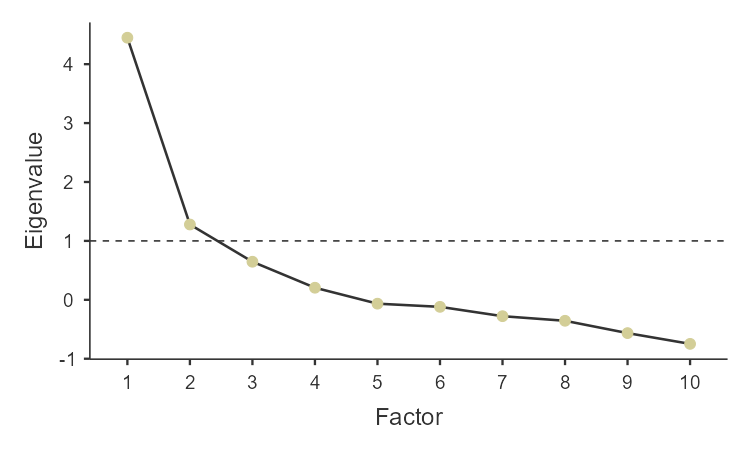


| **Supplementary Table S-2b: Internal consistency for ten items in the gambling sub-section of STEPS-IDD tool** | | |
| --- | --- | --- |
| **GDHG scale** | **Cronbach's Alpha value** | **Cronbach's Alpha if item deleted** |
| Complete scale | 0.872 | N/A |
| Item 1 | N/A | 0.860 |
| Item 2 | N/A | 0.871 |
| Item 3 | N/A | 0.877 |
| Item 4 | N/A | 0.851 |
| Item 5 | N/A | 0.834 |
| Item 6 | N/A | 0.862 |
| Item 7 | N/A | 0.877 |
| Item 8 | N/A | 0.837 |
| Item 9 | N/A | 0.837 |
| Item 10 | N/A | 0.870 |
| N/A: Not applicable | | |

**Supplementary Table S-3b: Results of Pearson and Spearman correlational analyses between the scores obtained on the STEPS-IDD gambling sub-section and the modified version of GDHGS for gambling**

| Correlation Matrix | | | | | | | |
| --- | --- | --- | --- | --- | --- | --- | --- |
|  | |  | | **Gambling_STEPS_Total** | | **Gambling_GDGHS_Total** | |
| **Gambling_STEPS_Total** |  | Pearson's r |  | — |  |  |  |
|  |  | df |  | — |  |  |  |
|  |  | p-value |  | — |  |  |  |
|  |  | Spearman's rho |  | — |  |  |  |
|  |  | df |  | — |  |  |  |
|  |  | p-value |  | — |  |  |  |
| **Gambling_GDGHS_Total** |  | Pearson's r |  | **0.769** | *** | — |  |
|  |  | df |  | 110 |  | — |  |
|  |  | p-value |  | < .001 |  | — |  |
|  |  | Spearman's rho |  | **0.737** | *** | — |  |
|  |  | df |  | 110 |  | — |  |
|  |  | p-value |  | < .001 |  | — |  |
| Note. * p < .05, ** p < .01, *** p < .001 | | | | | | | |
|  | | | | | | | |

**Supplementary Table S-4b: Frequency distribution table for total score on gambling sub-section of STEPS-IDD**

| **Gambling_STEPS_Total** | | | | | |
| --- | --- | --- | --- | --- | --- |
|  | | Frequency | Percent | Valid Percent | Cumulative Percent |
| Valid | 10 | 99 | 88.4 | 88.4 | 88.4 |
|  | 11 | 2 | 1.8 | 1.8 | 90.2 |
|  | 12 | 3 | 2.7 | 2.7 | 92.9 |
|  | 13 | 1 | .9 | .9 | 93.8 |
|  | 15 | 1 | .9 | .9 | 94.6 |
|  | 17 | 2 | 1.8 | 1.8 | 96.4 |
|  | 20 | 1 | .9 | .9 | 97.3 |
|  | 21 | 1 | .9 | .9 | 98.2 |
|  | 22 | 1 | .9 | .9 | 99.1 |
|  | 23 | 1 | .9 | .9 | 100.0 |
|  | Total | 112 | 100.0 | 100.0 |  |

**Supplementary Table S-5b: Summary output of the Receiver Operating Characteristic (ROC) analysis for the gambling sub-section of STEPS-IDD**

| Scale: Gambling_STEPS_Total | | | | | | | | | | | | | | | |
| --- | --- | --- | --- | --- | --- | --- | --- | --- | --- | --- | --- | --- | --- | --- | --- |
| **Cutpoint** | | **Sensitivity (%)** | | **Specificity (%)** | | **PPV (%)** | | **NPV (%)** | | **Youden's index** | | **AUC** | | **Metric Score** | |
| 10 |  | 100% |  | 0% |  | 2.68% |  | NaN% |  | 0.000 |  | 1.00 |  | 0.0268 |  |
| 11 |  | 100% |  | 90.83% |  | 23.08% |  | 100% |  | 0.908 |  | 1.00 |  | 0.9107 |  |
| 12 |  | 100% |  | 92.66% |  | 27.27% |  | 100% |  | 0.927 |  | 1.00 |  | 0.9286 |  |
| 13 |  | 100% |  | 95.41% |  | 37.5% |  | 100% |  | 0.954 |  | 1.00 |  | 0.9554 |  |
| 15 |  | 100% |  | 96.33% |  | 42.86% |  | 100% |  | 0.963 |  | 1.00 |  | 0.9643 |  |
| 17 |  | 100% |  | 97.25% |  | 50% |  | 100% |  | 0.972 |  | 1.00 |  | 0.9732 |  |
| 20 |  | 100% |  | 99.08% |  | 75% |  | 100% |  | 0.991 |  | 1.00 |  | 0.9911 |  |
| 21 |  | 100% |  | 100% |  | 100% |  | 100% |  | 1.000 |  | 1.00 |  | 1.0000 |  |
| 22 |  | 66.67% |  | 100% |  | 100% |  | 99.09% |  | 0.667 |  | 1.00 |  | 0.9911 |  |
| 23 |  | 33.33% |  | 100% |  | 100% |  | 98.2% |  | 0.333 |  | 1.00 |  | 0.9821 |  |
|  | | | | | | | | | | | | | | | |

**Supplementary Figure S-5b: Receiver Operating Characteristic (ROC) curve for the gambling sub-section of STEPS-IDD**


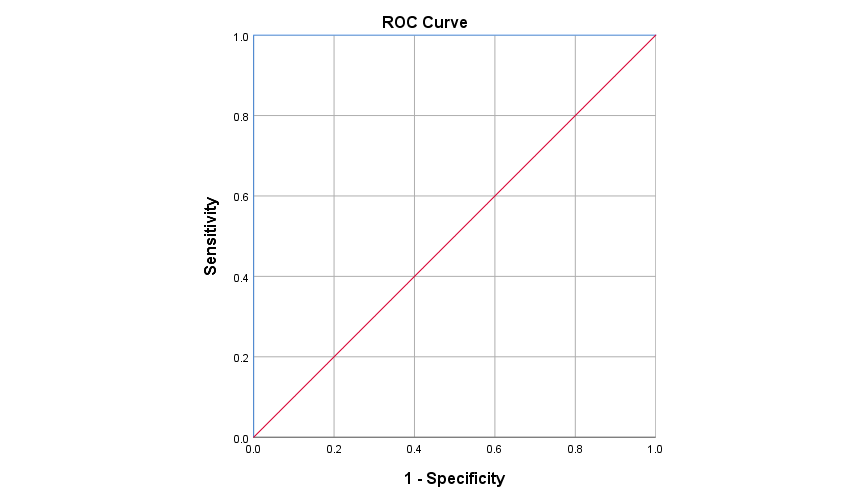


**Results of Exploratory Factor Analysis for social media use sub-section of the STEPS-IDD tool:**

Kaiser-Meyer-Olkin (KMO) coefficient= 0.88

Bartlett's Test (BT) of sphericity revealed significant p-value (<0.001)

**Supplementary Table S-1c: Summary of results obtained from exploratory factor analysis (EFA) on nine items of the STEPS-IDD sub-section for social media use**

| **Item**^#^ | **Factor Loadings ^a,b^** | | **Uniqueness** |
| --- | --- | --- | --- |
|  | **Factor 1** | **Factor 2** |  |
| 1 | 0.625 | - | 0.610 |
| 2 | 0.639 | - | 0.592 |
| 3 | 0.690 | - | 0.523 |
| 4 | 0.801 | - | 0.358 |
| 5 | 0.681 | - | 0.536 |
| 6 | 0.580 | - | 0.663 |
| 7 | 0.730 | - | 0.466 |
| 9 | 0.599 | - | 0.641 |
| 10 | 0.667 | - | 0.555 |
|  | ^a^ Percentage of the Total Variance Explained = 45.1%. ^b^ Only one factor was possible to be extracted from the EFA.  ^#^ Item 8 is included only for gambling sub-section of STEPS-IDD based on the ICD-11 criteria. | | |

**Supplementary Figure S1-c: Scree Plot for exploratory factor analysis (EFA) of the STEPS-IDD sub-section items for social media use**

### Scree Plot


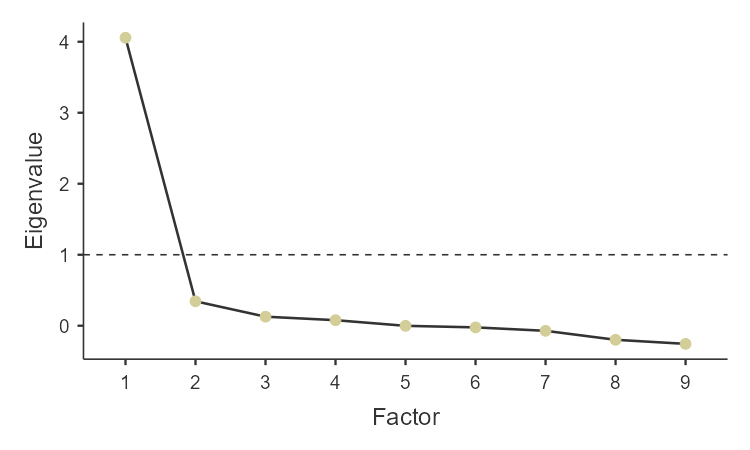


| **Supplementary Table S-2c: Internal consistency for nine items in the social media use sub-section of STEPS-IDD tool** | | |
| --- | --- | --- |
| **GDHG scale** | **Cronbach's Alpha value** | **Cronbach's Alpha if item deleted** |
| Complete scale | 0.879 | N/A |
| Item 1 | N/A | 0.869 |
| Item 2 | N/A | 0.868 |
| Item 3 | N/A | 0.865 |
| Item 4 | N/A | 0.855 |
| Item 5 | N/A | 0.865 |
| Item 6 | N/A | 0.873 |
| Item 7 | N/A | 0.861 |
| Item 9 | N/A | 0.872 |
| Item 10 | N/A | 0.866 |
| N/A: Not applicable | | |

**Supplementary Table S-3c: Results of Pearson and Spearman correlational analyses between the scores obtained on the STEPS-IDD social media use (SMU) sub-section and the modified version of GDHGS for social media use**

| Correlation Matrix | | | | | | | |
| --- | --- | --- | --- | --- | --- | --- | --- |
|  | |  | | **SMU_GDGHS_Total** | | **SMU_STEPS_Total** | |
| **SMU_GDGHS_Total** |  | Pearson's r |  | — |  |  |  |
|  |  | df |  | — |  |  |  |
|  |  | p-value |  | — |  |  |  |
|  |  | Spearman's rho |  | — |  |  |  |
|  |  | df |  | — |  |  |  |
|  |  | p-value |  | — |  |  |  |
| **SMU_STEPS_Total** |  | Pearson's r |  | **0.470** | *** | — |  |
|  |  | df |  | 110 |  | — |  |
|  |  | p-value |  | < .001 |  | — |  |
|  |  | Spearman's rho |  | **0.443** | *** | — |  |
|  |  | df |  | 110 |  | — |  |
|  |  | p-value |  | < .001 |  | — |  |
| Note. * p < .05, ** p < .01, *** p < .001 | | | | | | | |
|  | | | | | | | |

**Supplementary Table S-4c: Frequency distribution table for total score on social media use (SMU) sub-section of STEPS-IDD**

| **SMU_STEPS_Total_Minus_Q9** | | | | | | |
| --- | --- | --- | --- | --- | --- | --- |
|  | | **Frequency** | **Percent** | **Valid Percent** | **Cumulative Percent** |  |
| Valid | 9 | 2 | 1.8 | 1.8 | 1.8 |  |
|  | 10 | 1 | .9 | .9 | 2.7 |  |
|  | 11 | 1 | .9 | .9 | 3.6 |  |
|  | 12 | 4 | 3.6 | 3.6 | 7.1 |  |
|  | 13 | 5 | 4.5 | 4.5 | 11.6 |  |
|  | 14 | 5 | 4.5 | 4.5 | 16.1 |  |
|  | 15 | 4 | 3.6 | 3.6 | 19.6 |  |
|  | 16 | 9 | 8.0 | 8.0 | 27.7 |  |
|  | 17 | 7 | 6.3 | 6.3 | 33.9 |  |
|  | 18 | 5 | 4.5 | 4.5 | 38.4 |  |
|  | 19 | 6 | 5.4 | 5.4 | 43.8 |  |
|  | 20 | 5 | 4.5 | 4.5 | 48.2 |  |
|  | 21 | 3 | 2.7 | 2.7 | 50.9 |  |
|  | 22 | 4 | 3.6 | 3.6 | 54.5 |  |
|  | 23 | 15 | 13.4 | 13.4 | 67.9 |  |
|  | 24 | 5 | 4.5 | 4.5 | 72.3 |  |
|  | 25 | 4 | 3.6 | 3.6 | 75.9 |  |
|  | 26 | 4 | 3.6 | 3.6 | 79.5 |  |
|  | 27 | 1 | .9 | .9 | 80.4 |  |
|  | 28 | 6 | 5.4 | 5.4 | 85.7 |  |
|  | 29 | 2 | 1.8 | 1.8 | 87.5 |  |
|  | 30 | 3 | 2.7 | 2.7 | 90.2 |  |
|  | 31 | 2 | 1.8 | 1.8 | 92.0 |  |
|  | 32 | 5 | 4.5 | 4.5 | 96.4 |  |
|  | 33 | 2 | 1.8 | 1.8 | 98.2 |  |
|  | 34 | 1 | .9 | .9 | 99.1 |  |
|  | 35 | 1 | .9 | .9 | 100.0 |  |
|  | Total | 112 | 100.0 | 100.0 |  |  |

**Supplementary Table S-5c: Summary output of the Receiver Operating Characteristic (ROC) analysis for the social media use (SMU) sub-section of STEPS-IDD**

| Scale: SMU_STEPS_Total | | | | | | | | | | | | | | | |
| --- | --- | --- | --- | --- | --- | --- | --- | --- | --- | --- | --- | --- | --- | --- | --- |
| **Cutpoint** | | **Sensitivity (%)** | | **Specificity (%)** | | **PPV (%)** | | **NPV (%)** | | **Youden's index** | | **AUC** | | **Metric Score** | |
| 9 |  | 100% |  | 0% |  | 44.64% |  | NaN% |  | 0.00000 |  | 0.773 |  | 0.446 |  |
| 10 |  | 100% |  | 3.23% |  | 45.45% |  | 100% |  | 0.03226 |  | 0.773 |  | 0.464 |  |
| 11 |  | 100% |  | 4.84% |  | 45.87% |  | 100% |  | 0.04839 |  | 0.773 |  | 0.473 |  |
| 12 |  | 100% |  | 6.45% |  | 46.3% |  | 100% |  | 0.06452 |  | 0.773 |  | 0.482 |  |
| 13 |  | 98% |  | 11.29% |  | 47.12% |  | 87.5% |  | 0.09290 |  | 0.773 |  | 0.500 |  |
| 14 |  | 96% |  | 17.74% |  | 48.48% |  | 84.62% |  | 0.13742 |  | 0.773 |  | 0.527 |  |
| 15 |  | 94% |  | 24.19% |  | 50% |  | 83.33% |  | 0.18194 |  | 0.773 |  | 0.554 |  |
| 16 |  | 92% |  | 29.03% |  | 51.11% |  | 81.82% |  | 0.21032 |  | 0.773 |  | 0.571 |  |
| 17 |  | 88% |  | 40.32% |  | 54.32% |  | 80.65% |  | 0.28323 |  | 0.773 |  | 0.616 |  |
| 18 |  | 84% |  | 48.39% |  | 56.76% |  | 78.95% |  | 0.32387 |  | 0.773 |  | 0.643 |  |
| 19 |  | 80% |  | 53.23% |  | 57.97% |  | 76.74% |  | 0.33226 |  | 0.773 |  | 0.652 |  |
| 20 |  | 78% |  | 61.29% |  | 61.9% |  | 77.55% |  | 0.39290 |  | 0.773 |  | 0.688 |  |
| 21 |  | 74% |  | 66.13% |  | 63.79% |  | 75.93% |  | 0.40129 |  | 0.773 |  | 0.696 |  |
| 22 |  | 72% |  | 69.35% |  | 65.45% |  | 75.44% |  | 0.41355 |  | 0.773 |  | 0.705 |  |
| 23 |  | 68% |  | 72.58% |  | 66.67% |  | 73.77% |  | 0.40581 |  | 0.773 |  | 0.705 |  |
| 24 |  | 58% |  | 88.71% |  | 80.56% |  | 72.37% |  | 0.46710 |  | 0.773 |  | 0.750 |  |
| 25 |  | 50% |  | 90.32% |  | 80.65% |  | 69.14% |  | 0.40323 |  | 0.773 |  | 0.723 |  |
| 26 |  | 46% |  | 93.55% |  | 85.19% |  | 68.24% |  | 0.39548 |  | 0.773 |  | 0.723 |  |
| 27 |  | 38% |  | 93.55% |  | 82.61% |  | 65.17% |  | 0.31548 |  | 0.773 |  | 0.688 |  |
| 28 |  | 36% |  | 93.55% |  | 81.82% |  | 64.44% |  | 0.29548 |  | 0.773 |  | 0.679 |  |
| 29 |  | 24% |  | 93.55% |  | 75% |  | 60.42% |  | 0.17548 |  | 0.773 |  | 0.625 |  |
| 30 |  | 22% |  | 95.16% |  | 78.57% |  | 60.2% |  | 0.17161 |  | 0.773 |  | 0.625 |  |
| 31 |  | 18% |  | 96.77% |  | 81.82% |  | 59.41% |  | 0.14774 |  | 0.773 |  | 0.616 |  |
| 32 |  | 14% |  | 96.77% |  | 77.78% |  | 58.25% |  | 0.10774 |  | 0.773 |  | 0.598 |  |
| 33 |  | 6% |  | 98.39% |  | 75% |  | 56.48% |  | 0.04387 |  | 0.773 |  | 0.571 |  |
| 34 |  | 2% |  | 98.39% |  | 50% |  | 55.45% |  | 0.00387 |  | 0.773 |  | 0.554 |  |
| 35 |  | 2% |  | 100% |  | 100% |  | 55.86% |  | 0.02000 |  | 0.773 |  | 0.563 |  |
|  | | | | | | | | | | | | | | | |

**Supplementary Figure S-5c: Receiver Operating Characteristic (ROC) curve for the social media use (SMU) sub-section of STEPS-IDD**


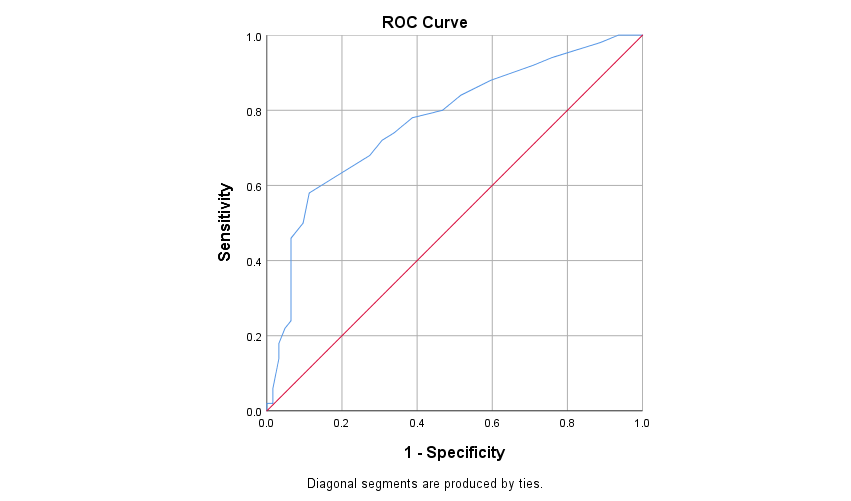


**Results of Exploratory Factor Analysis for Shopping or Buying sub-section of the STEPS-IDD tool:**

Kaiser-Meyer-Olkin (KMO) coefficient= 0.81

Bartlett's Test (BT) of sphericity revealed significant p-value (<0.001)

**Supplementary Table S-1d: Summary of results obtained from exploratory factor analysis (EFA) on nine items of the STEPS-IDD sub-section for Shopping or Buying**

| **Item^#^** | **Factor Loadings ^a,b^** | | **Uniqueness** |
| --- | --- | --- | --- |
|  | **Factor 1** | **Factor 2** |  |
| 1 | 0.642 | - | 0.588 |
| 2 | 0.739 | - | 0.453 |
| 3 | 0.752 | - | 0.434 |
| 4 | 0.723 | - | 0.477 |
| 5 | 0.691 | - | 0.523 |
| 6 | 0.700 | - | 0.510 |
| 7 | 0.615 | - | 0.622 |
| 9 | 0.573 | - | 0.672 |
| 10 | 0.659 | - | 0.566 |
|  | ^a^ Percentage of the Total Variance Explained = 46.2%. ^b^ Only one factor was possible to be extracted from the EFA.  ^#^ Item 8 is included only for gambling sub-section of STEPS-IDD based on the ICD-11 criteria. | | |

**Supplementary Figure S1-d: Scree Plot for exploratory factor analysis (EFA) of the STEPS-IDD sub-section items for Shopping or Buying**

### Scree Plot


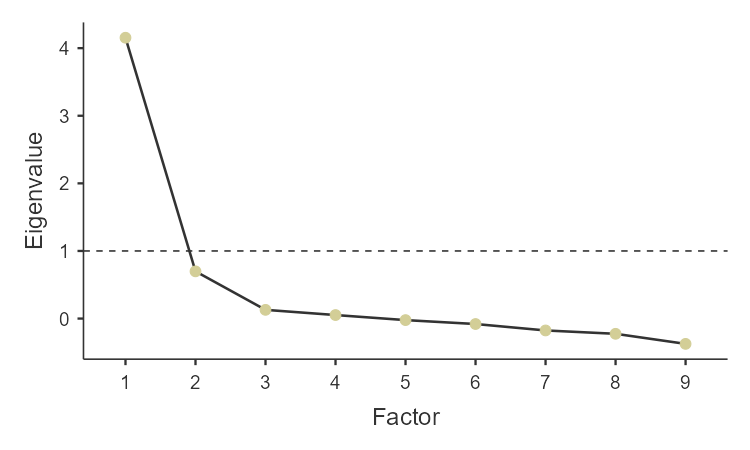


| **Supplementary Table S-2d: Internal consistency for nine items in the Shopping or Buying sub-section of STEPS-IDD tool** | | |
| --- | --- | --- |
| **GDHG scale** | **Cronbach's Alpha value** | **Cronbach's Alpha if item deleted** |
| Complete scale | 0.879 | N/A |
| Item 1 | N/A | 0.869 |
| Item 2 | N/A | 0.858 |
| Item 3 | N/A | 0.859 |
| Item 4 | N/A | 0.859 |
| Item 5 | N/A | 0.864 |
| Item 6 | N/A | 0.866 |
| Item 7 | N/A | 0.871 |
| Item 9 | N/A | 0.874 |
| Item 10 | N/A | 0.868 |
| N/A: Not applicable | | |

**Supplementary Table S-3d: Results of Pearson and Spearman correlational analyses between the scores obtained on the STEPS-IDD Shopping or Buying sub-section and the modified version of GDHGS for Shopping or Buying**

| Correlation Matrix | | | | | | | |
| --- | --- | --- | --- | --- | --- | --- | --- |
|  | |  | | **Shopping/Buying_GDHGS_Total** | | **Shopping/Buying_STEPS_Total** | |
| **Shopping/Buying_GDHGS_Total** |  | Pearson's r |  | — |  |  |  |
|  |  | df |  | — |  |  |  |
|  |  | p-value |  | — |  |  |  |
|  |  | Spearman's rho |  | — |  |  |  |
|  |  | df |  | — |  |  |  |
|  |  | p-value |  | — |  |  |  |
| **Shopping/Buying_STEPS_Total** |  | Pearson's r |  | **0.716** | *** | — |  |
|  |  | df |  | 110 |  | — |  |
|  |  | p-value |  | < 0.001 |  | — |  |
|  |  | Spearman's rho |  | **0.595** | *** | — |  |
|  |  | df |  | 110 |  | — |  |
|  |  | p-value |  | < 0.001 |  | — |  |
| Note. * p < .05, ** p < .01, *** p < .001 | | | | | | | |
|  | | | | | | | |

**Supplementary Table S-4d: Frequency distribution table for total score on Shopping/ Buying sub-section of STEPS-IDD**

| **ShoppingBuying_STEPS_Total** | | | | | | |
| --- | --- | --- | --- | --- | --- | --- |
|  | | **Frequency** | **Percent** | **Valid Percent** | **Cumulative Percent** |  |
| Valid | 9 | 33 | 29.5 | 29.5 | 29.5 |  |
|  | 10 | 12 | 10.7 | 10.7 | 40.2 |  |
|  | 11 | 12 | 10.7 | 10.7 | 50.9 |  |
|  | 12 | 13 | 11.6 | 11.6 | 62.5 |  |
|  | 13 | 4 | 3.6 | 3.6 | 66.1 |  |
|  | 14 | 9 | 8.0 | 8.0 | 74.1 |  |
|  | 15 | 4 | 3.6 | 3.6 | 77.7 |  |
|  | 16 | 3 | 2.7 | 2.7 | 80.4 |  |
|  | 17 | 3 | 2.7 | 2.7 | 83.0 |  |
|  | 18 | 5 | 4.5 | 4.5 | 87.5 |  |
|  | 19 | 3 | 2.7 | 2.7 | 90.2 |  |
|  | 20 | 1 | .9 | .9 | 91.1 |  |
|  | 21 | 3 | 2.7 | 2.7 | 93.8 |  |
|  | 22 | 1 | .9 | .9 | 94.6 |  |
|  | 23 | 2 | 1.8 | 1.8 | 96.4 |  |
|  | 24 | 3 | 2.7 | 2.7 | 99.1 |  |
|  | 34 | 1 | .9 | .9 | 100.0 |  |
|  | Total | 112 | 100.0 | 100.0 |  |  |

**Supplementary Table S-5d: Summary output of the Receiver Operating Characteristic (ROC) analysis for the Shopping/ Buying sub-section of STEPS-IDD**

| Scale: Shopping/Buying_STEPS_Total | | | | | | | | | | | | | | | |
| --- | --- | --- | --- | --- | --- | --- | --- | --- | --- | --- | --- | --- | --- | --- | --- |
| **Cutpoint** | | **Sensitivity (%)** | | **Specificity (%)** | | **PPV (%)** | | **NPV (%)** | | **Youden's index** | | **AUC** | | **Metric Score** | |
| 9 |  | 100% |  | 0% |  | 6.25% |  | NaN% |  | 0.000 |  | 0.902 |  | 0.0625 |  |
| 10 |  | 100% |  | 31.43% |  | 8.86% |  | 100% |  | 0.314 |  | 0.902 |  | 0.3571 |  |
| 11 |  | 100% |  | 42.86% |  | 10.45% |  | 100% |  | 0.429 |  | 0.902 |  | 0.4643 |  |
| 12 |  | 100% |  | 54.29% |  | 12.73% |  | 100% |  | 0.543 |  | 0.902 |  | 0.5714 |  |
| 13 |  | 100% |  | 66.67% |  | 16.67% |  | 100% |  | 0.667 |  | 0.902 |  | 0.6875 |  |
| 14 |  | 85.71% |  | 69.52% |  | 15.79% |  | 98.65% |  | 0.552 |  | 0.902 |  | 0.7054 |  |
| 15 |  | 71.43% |  | 77.14% |  | 17.24% |  | 97.59% |  | 0.486 |  | 0.902 |  | 0.7679 |  |
| 16 |  | 71.43% |  | 80.95% |  | 20% |  | 97.7% |  | 0.524 |  | 0.902 |  | 0.8036 |  |
| 17 |  | 71.43% |  | 83.81% |  | 22.73% |  | 97.78% |  | 0.552 |  | 0.902 |  | 0.8304 |  |
| 18 |  | 71.43% |  | 86.67% |  | 26.32% |  | 97.85% |  | 0.581 |  | 0.902 |  | 0.8571 |  |
| 19 |  | 71.43% |  | 91.43% |  | 35.71% |  | 97.96% |  | 0.629 |  | 0.902 |  | 0.9018 |  |
| 20 |  | 57.14% |  | 93.33% |  | 36.36% |  | 97.03% |  | 0.505 |  | 0.902 |  | 0.9107 |  |
| 21 |  | 57.14% |  | 94.29% |  | 40% |  | 97.06% |  | 0.514 |  | 0.902 |  | 0.9196 |  |
| 22 |  | 57.14% |  | 97.14% |  | 57.14% |  | 97.14% |  | 0.543 |  | 0.902 |  | 0.9464 |  |
| 23 |  | 57.14% |  | 98.1% |  | 66.67% |  | 97.17% |  | 0.552 |  | 0.902 |  | 0.9554 |  |
| 24 |  | 42.86% |  | 99.05% |  | 75% |  | 96.3% |  | 0.419 |  | 0.902 |  | 0.9554 |  |
| 34 |  | 14.29% |  | 100% |  | 100% |  | 94.59% |  | 0.143 |  | 0.902 |  | 0.9464 |  |
|  | | | | | | | | | | | | | | | |

**Supplementary Figure S-5d: Receiver Operating Characteristic (ROC) curve for the Shopping/ Buying sub-section of STEPS-IDD**


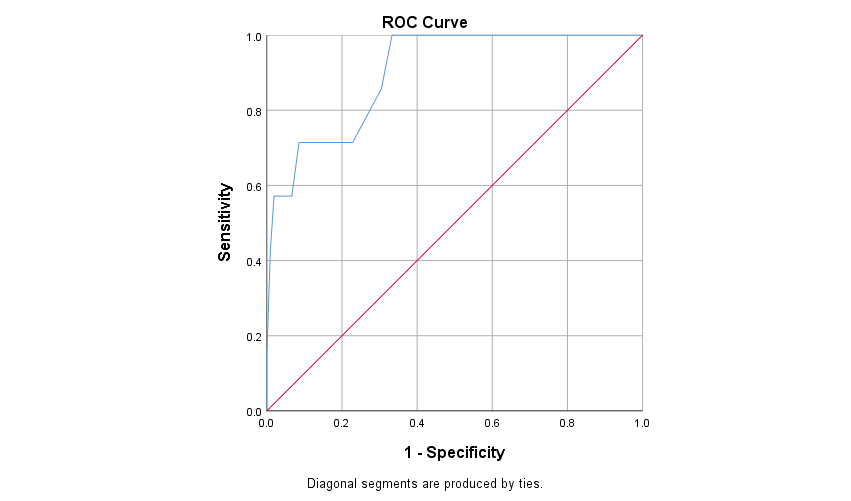


**Results of Exploratory Factor Analysis for** **OTT viewing sub-section of the STEPS-IDD tool:**

Kaiser-Meyer-Olkin (KMO) coefficient= 0.89

Bartlett's Test (BT) of sphericity revealed significant p-value (<0.001)

**Supplementary Table S-1e: Summary of results obtained from exploratory factor analysis (EFA) on nine items of the STEPS-IDD sub-section for OTT viewing**

| **Item^#^** | **Factor Loadings ^a,b^** | | **Uniqueness** |
| --- | --- | --- | --- |
|  | **Factor 1** | **Factor 2** |  |
| 1 | 0.530 | - | 0.719 |
| 2 | 0.760 | - | 0.423 |
| 3 | 0.703 | - | 0.506 |
| 4 | 0.783 | - | 0.387 |
| 5 | 0.623 | - | 0.612 |
| 6 | 0.682 | - | 0.535 |
| 7 | 0.701 | - | 0.509 |
| 9 | 0.726 | - | 0.472 |
| 10 | 0.725 | - | 0.475 |
|  | ^a^ Percentage of the Total Variance Explained = 48.5%. ^b^ Only one factor was possible to be extracted from the EFA.  ^#^ Item 8 is included only for gambling sub-section of STEPS-IDD based on the ICD-11 criteria. | | |

**Supplementary Figure S1-e: Scree Plot for exploratory factor analysis (EFA) of the STEPS-IDD sub-section items for OTT viewing**

### Scree Plot


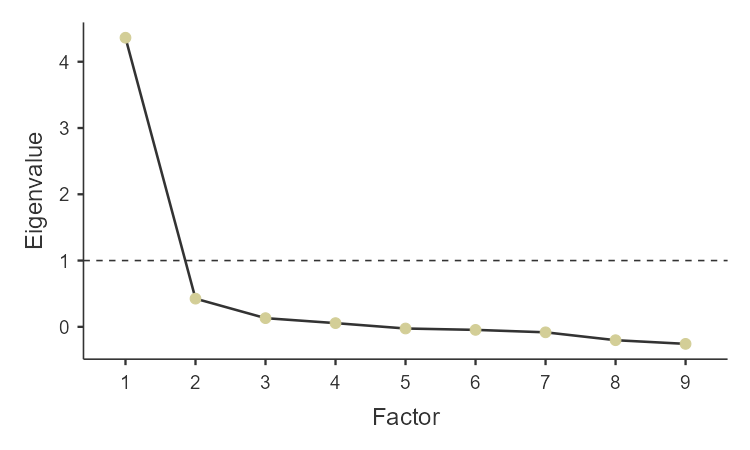


| **Supplementary Table S-2e: Internal consistency for nine items in the OTT viewing sub-section of STEPS-IDD tool** | | |
| --- | --- | --- |
| **GDHG scale** | **Cronbach's Alpha value** | **Cronbach's Alpha if item deleted** |
| Complete scale | 0.891 | N/A |
| Item 1 | N/A | 0.891 |
| Item 2 | N/A | 0.873 |
| Item 3 | N/A | 0.879 |
| Item 4 | N/A | 0.872 |
| Item 5 | N/A | 0.884 |
| Item 6 | N/A | 0.881 |
| Item 7 | N/A | 0.879 |
| Item 9 | N/A | 0.878 |
| Item 10 | N/A | 0.878 |
| N/A: Not applicable | | |

**Supplementary Table S-3e: Results of Pearson and Spearman correlational analyses between the scores obtained on the STEPS-IDD OTT viewing sub-section and the modified version of GDHGS for OTT viewing**

| Correlation Matrix | | | | | | | |
| --- | --- | --- | --- | --- | --- | --- | --- |
|  | |  | | **OTTviewing_STEPS_Total** | | **OTTviewing_GDHGS_Total** | |
| **OTTviewing_STEPS_Total** |  | Pearson's r |  | — |  |  |  |
|  |  | df |  | — |  |  |  |
|  |  | p-value |  | — |  |  |  |
|  |  | Spearman's rho |  | — |  |  |  |
|  |  | df |  | — |  |  |  |
|  |  | p-value |  | — |  |  |  |
| **OTTviewing_GDHGS_Total** |  | Pearson's r |  | **0.625** | *** | — |  |
|  |  | df |  | 110 |  | — |  |
|  |  | p-value |  | < 0.001 |  | — |  |
|  |  | Spearman's rho |  | **0.601** | *** | — |  |
|  |  | df |  | 110 |  | — |  |
|  |  | p-value |  | < 0.001 |  | — |  |
| Note. * p < .05, ** p < .01, *** p < .001 | | | | | | | |
|  | | | | | | | |

**Supplementary Table S-4e: Frequency distribution table for total score on OTT watching sub-section of STEPS-IDD**

| **OTTviewing_STEPS_Total_Minus_Q9** | | | | | | |
| --- | --- | --- | --- | --- | --- | --- |
|  | | **Frequency** | **Percent** | **Valid Percent** | **Cumulative Percent** |  |
| Valid | 9 | 5 | 4.5 | 4.5 | 4.5 |  |
|  | 10 | 6 | 5.4 | 5.4 | 9.8 |  |
|  | 11 | 3 | 2.7 | 2.7 | 12.5 |  |
|  | 12 | 4 | 3.6 | 3.6 | 16.1 |  |
|  | 13 | 3 | 2.7 | 2.7 | 18.8 |  |
|  | 14 | 5 | 4.5 | 4.5 | 23.2 |  |
|  | 15 | 8 | 7.1 | 7.1 | 30.4 |  |
|  | 16 | 5 | 4.5 | 4.5 | 34.8 |  |
|  | 17 | 8 | 7.1 | 7.1 | 42.0 |  |
|  | 18 | 10 | 8.9 | 8.9 | 50.9 |  |
|  | 19 | 6 | 5.4 | 5.4 | 56.3 |  |
|  | 20 | 8 | 7.1 | 7.1 | 63.4 |  |
|  | 21 | 4 | 3.6 | 3.6 | 67.0 |  |
|  | 22 | 8 | 7.1 | 7.1 | 74.1 |  |
|  | 23 | 3 | 2.7 | 2.7 | 76.8 |  |
|  | 24 | 3 | 2.7 | 2.7 | 79.5 |  |
|  | 25 | 4 | 3.6 | 3.6 | 83.0 |  |
|  | 27 | 2 | 1.8 | 1.8 | 84.8 |  |
|  | 28 | 3 | 2.7 | 2.7 | 87.5 |  |
|  | 29 | 4 | 3.6 | 3.6 | 91.1 |  |
|  | 31 | 4 | 3.6 | 3.6 | 94.6 |  |
|  | 32 | 2 | 1.8 | 1.8 | 96.4 |  |
|  | 33 | 2 | 1.8 | 1.8 | 98.2 |  |
|  | 34 | 1 | .9 | .9 | 99.1 |  |
|  | 35 | 1 | .9 | .9 | 100.0 |  |
|  | Total | 112 | 100.0 | 100.0 |  |  |

**Supplementary Table S-5e: Summary output of the Receiver Operating Characteristic (ROC) analysis for the OTT viewing sub-section of STEPS-IDD**

| Scale: OTTviewing_STEPS_Total | | | | | | | | | | | | | | | |
| --- | --- | --- | --- | --- | --- | --- | --- | --- | --- | --- | --- | --- | --- | --- | --- |
| **Cutpoint** | | **Sensitivity (%)** | | **Specificity (%)** | | **PPV (%)** | | **NPV (%)** | | **Youden's index** | | **AUC** | | **Metric Score** | |
| 9 |  | 100% |  | 0% |  | 29.46% |  | NaN% |  | 0.0000 |  | 0.754 |  | 0.295 |  |
| 10 |  | 96.97% |  | 5.06% |  | 29.91% |  | 80% |  | 0.0203 |  | 0.754 |  | 0.321 |  |
| 11 |  | 96.97% |  | 12.66% |  | 31.68% |  | 90.91% |  | 0.0963 |  | 0.754 |  | 0.375 |  |
| 12 |  | 96.97% |  | 16.46% |  | 32.65% |  | 92.86% |  | 0.1343 |  | 0.754 |  | 0.402 |  |
| 13 |  | 96.97% |  | 21.52% |  | 34.04% |  | 94.44% |  | 0.1849 |  | 0.754 |  | 0.438 |  |
| 14 |  | 96.97% |  | 25.32% |  | 35.16% |  | 95.24% |  | 0.2229 |  | 0.754 |  | 0.464 |  |
| 15 |  | 96.97% |  | 31.65% |  | 37.21% |  | 96.15% |  | 0.2862 |  | 0.754 |  | 0.509 |  |
| 16 |  | 93.94% |  | 40.51% |  | 39.74% |  | 94.12% |  | 0.3445 |  | 0.754 |  | 0.563 |  |
| 17 |  | 93.94% |  | 46.84% |  | 42.47% |  | 94.87% |  | 0.4077 |  | 0.754 |  | 0.607 |  |
| 18 |  | 81.82% |  | 51.9% |  | 41.54% |  | 87.23% |  | 0.3372 |  | 0.754 |  | 0.607 |  |
| 19 |  | 66.67% |  | 58.23% |  | 40% |  | 80.7% |  | 0.2489 |  | 0.754 |  | 0.607 |  |
| 20 |  | 63.64% |  | 64.56% |  | 42.86% |  | 80.95% |  | 0.2819 |  | 0.754 |  | 0.643 |  |
| 21 |  | 57.58% |  | 72.15% |  | 46.34% |  | 80.28% |  | 0.2973 |  | 0.754 |  | 0.679 |  |
| 22 |  | 57.58% |  | 77.22% |  | 51.35% |  | 81.33% |  | 0.3479 |  | 0.754 |  | 0.714 |  |
| 23 |  | 51.52% |  | 84.81% |  | 58.62% |  | 80.72% |  | 0.3633 |  | 0.754 |  | 0.750 |  |
| 24 |  | 45.45% |  | 86.08% |  | 57.69% |  | 79.07% |  | 0.3153 |  | 0.754 |  | 0.741 |  |
| 25 |  | 45.45% |  | 89.87% |  | 65.22% |  | 79.78% |  | 0.3533 |  | 0.754 |  | 0.768 |  |
| 27 |  | 36.36% |  | 91.14% |  | 63.16% |  | 77.42% |  | 0.2750 |  | 0.754 |  | 0.750 |  |
| 28 |  | 36.36% |  | 93.67% |  | 70.59% |  | 77.89% |  | 0.3003 |  | 0.754 |  | 0.768 |  |
| 29 |  | 30.3% |  | 94.94% |  | 71.43% |  | 76.53% |  | 0.2524 |  | 0.754 |  | 0.759 |  |
| 31 |  | 21.21% |  | 96.2% |  | 70% |  | 74.51% |  | 0.1741 |  | 0.754 |  | 0.741 |  |
| 32 |  | 18.18% |  | 100% |  | 100% |  | 74.53% |  | 0.1818 |  | 0.754 |  | 0.759 |  |
| 33 |  | 12.12% |  | 100% |  | 100% |  | 73.15% |  | 0.1212 |  | 0.754 |  | 0.741 |  |
| 34 |  | 6.06% |  | 100% |  | 100% |  | 71.82% |  | 0.0606 |  | 0.754 |  | 0.723 |  |
| 35 |  | 3.03% |  | 100% |  | 100% |  | 71.17% |  | 0.0303 |  | 0.754 |  | 0.714 |  |
|  | | | | | | | | | | | | | | | |

**Supplementary Figure S-5e: Receiver Operating Characteristic (ROC) curve for the OTT viewing sub-section of STEPS-IDD**


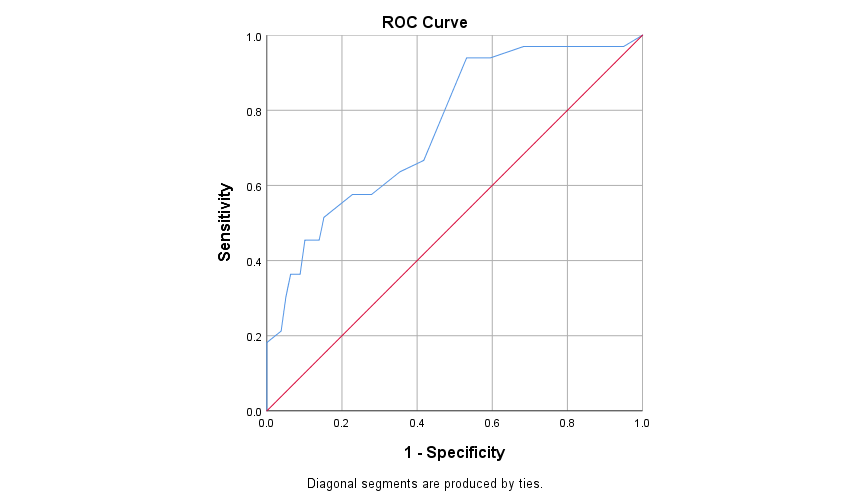


**Results of Exploratory Factor Analysis for Pornography watching sub-section of the STEPS-IDD tool:**

Kaiser-Meyer-Olkin (KMO) coefficient= 0.76

Bartlett's Test (BT) of sphericity revealed significant p-value (<0.001)

**Supplementary Table S-1f: Summary of results obtained from exploratory factor analysis (EFA) on nine items of the STEPS-IDD sub-section for pornography viewing**

| **Items^#^** | **Factor Loadings ^a,b^** | | **Uniqueness** |
| --- | --- | --- | --- |
|  | **Factor 1** | **Factor 2** |  |
| 1 | 0.557 | - | 0.690 |
| 2 | 0.606 | - | 0.633 |
| 3 | 0.570 | - | 0.675 |
| 4 | 0.837 | - | 0.299 |
| 5 | 0.628 | - | 0.606 |
| 6 | 0.851 | - | 0.275 |
| 7 | 0.504 | - | 0.746 |
| 9 | 0.631 | - | 0.602 |
| 10 | 0.860 | - | 0.260 |
|  | ^a^ Percentage of the Total Variance Explained = 46.8%. ^b^ Only one factor was possible to be extracted from the EFA.  ^#^ Item 8 is included only for gambling sub-section of STEPS-IDD based on the ICD-11 criteria. | | |

**Supplementary Figure S-1f: Scree Plot for exploratory factor analysis (EFA) of the STEPS-IDD sub-section items for gaming**

### Scree Plot


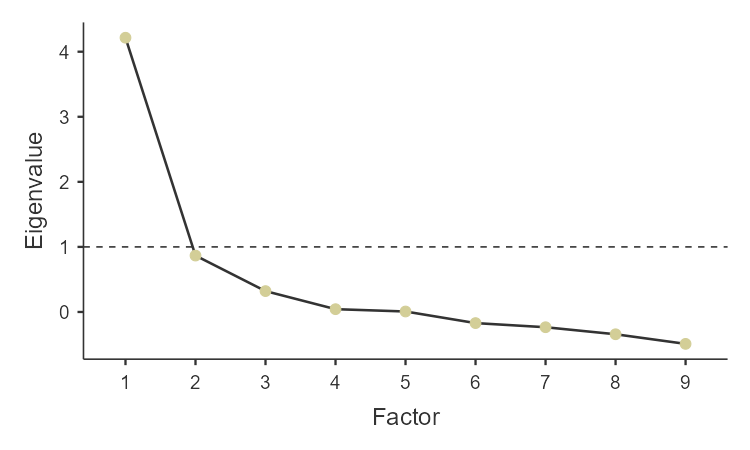


| **Supplementary Table S-2f: Internal consistency for nine items in the Pornography viewing sub-section of STEPS-IDD tool** | | |
| --- | --- | --- |
| **GDHG scale** | **Cronbach's Alpha value** | **Cronbach's Alpha if item deleted** |
| Complete scale | 0.865 | N/A |
| Item 1 | N/A | 0.866 |
| Item 2 | N/A | 0.848 |
| Item 3 | N/A | 0.852 |
| Item 4 | N/A | 0.838 |
| Item 5 | N/A | 0.851 |
| Item 6 | N/A | 0.839 |
| Item 7 | N/A | 0.864 |
| Item 9 | N/A | 0.856 |
| Item 10 | N/A | 0.839 |
| N/A: Not applicable | | |

**Supplementary Table S-3f: Results of Pearson and Spearman correlational analyses between the scores obtained on the STEPS-IDD Pornography viewing sub-section and the modified version of GDHGS for Pornography viewing**

| Correlation Matrix | | | | | | | |
| --- | --- | --- | --- | --- | --- | --- | --- |
|  | |  | | **Porn_GDHGS_Total** | | **Porn_STEPS_Total** | |
| **Porn_GDHGS_Total** |  | Pearson's r |  | — |  |  |  |
|  |  | df |  | — |  |  |  |
|  |  | p-value |  | — |  |  |  |
|  |  | Spearman's rho |  | — |  |  |  |
|  |  | df |  | — |  |  |  |
|  |  | p-value |  | — |  |  |  |
| **Porn_STEPS_Total** |  | Pearson's r |  | **0.607** *** | | — |  |
|  |  | df |  | 110 |  | — |  |
|  |  | p-value |  | < 0.001 |  | — |  |
|  |  | Spearman's rho |  | **0.645** *** | | — |  |
|  |  | df |  | 110 |  | — |  |
|  |  | p-value |  | < 0.001 |  | — |  |
| Note. * p < .05, ** p < .01, *** p < .001 | | | | | | | |
|  | | | | | | | |

**Supplementary Table S-4f: Frequency distribution table for total score on Pornography viewing sub-section of STEPS-IDD**

| **Porn_STEPS_Total** | | | | | | |
| --- | --- | --- | --- | --- | --- | --- |
|  | | **Frequency** | **Percent** | **Valid Percent** | **Cumulative Percent** |  |
| Valid | 9 | 80 | 71.4 | 71.4 | 71.4 |  |
|  | 10 | 12 | 10.7 | 10.7 | 82.1 |  |
|  | 11 | 8 | 7.1 | 7.1 | 89.3 |  |
|  | 12 | 2 | 1.8 | 1.8 | 91.1 |  |
|  | 13 | 3 | 2.7 | 2.7 | 93.8 |  |
|  | 14 | 2 | 1.8 | 1.8 | 95.5 |  |
|  | 15 | 1 | .9 | .9 | 96.4 |  |
|  | 16 | 1 | .9 | .9 | 97.3 |  |
|  | 17 | 1 | .9 | .9 | 98.2 |  |
|  | 20 | 1 | .9 | .9 | 99.1 |  |
|  | 23 | 1 | .9 | .9 | 100.0 |  |
|  | Total | 112 | 100.0 | 100.0 |  |  |

**Supplementary Table S-5f: Summary output of the Receiver Operating Characteristic (ROC) analysis for the Pornography watching sub-section of STEPS-IDD**

| Scale: Porn_STEPS_Total | | | | | | | | | | | | | | | |
| --- | --- | --- | --- | --- | --- | --- | --- | --- | --- | --- | --- | --- | --- | --- | --- |
| **Cutpoint** | | **Sensitivity (%)** | | **Specificity (%)** | | **PPV (%)** | | **NPV (%)** | | **Youden's index** | | **AUC** | | **Metric Score** | |
| 9 |  | 100% |  | 0% |  | 5.36% |  | NaN% |  | 0.000 |  | 0.839 |  | 0.0536 |  |
| 10 |  | 83.33% |  | 74.53% |  | 15.62% |  | 98.75% |  | 0.579 |  | 0.839 |  | 0.7500 |  |
| 11 |  | 66.67% |  | 84.91% |  | 20% |  | 97.83% |  | 0.516 |  | 0.839 |  | 0.8393 |  |
| 12 |  | 50% |  | 91.51% |  | 25% |  | 97% |  | 0.415 |  | 0.839 |  | 0.8929 |  |
| 13 |  | 50% |  | 93.4% |  | 30% |  | 97.06% |  | 0.434 |  | 0.839 |  | 0.9107 |  |
| 14 |  | 50% |  | 96.23% |  | 42.86% |  | 97.14% |  | 0.462 |  | 0.839 |  | 0.9375 |  |
| 15 |  | 50% |  | 98.11% |  | 60% |  | 97.2% |  | 0.481 |  | 0.839 |  | 0.9554 |  |
| 16 |  | 50% |  | 99.06% |  | 75% |  | 97.22% |  | 0.491 |  | 0.839 |  | 0.9643 |  |
| 17 |  | 33.33% |  | 99.06% |  | 66.67% |  | 96.33% |  | 0.324 |  | 0.839 |  | 0.9554 |  |
| 20 |  | 16.67% |  | 99.06% |  | 50% |  | 95.45% |  | 0.157 |  | 0.839 |  | 0.9464 |  |
| 23 |  | 16.67% |  | 100% |  | 100% |  | 95.5% |  | 0.167 |  | 0.839 |  | 0.9554 |  |
|  | | | | | | | | | | | | | | | |

**Supplementary Figure S-5f: Receiver Operating Characteristic (ROC) curve for the Pornography viewing sub-section of STEPS-IDD**


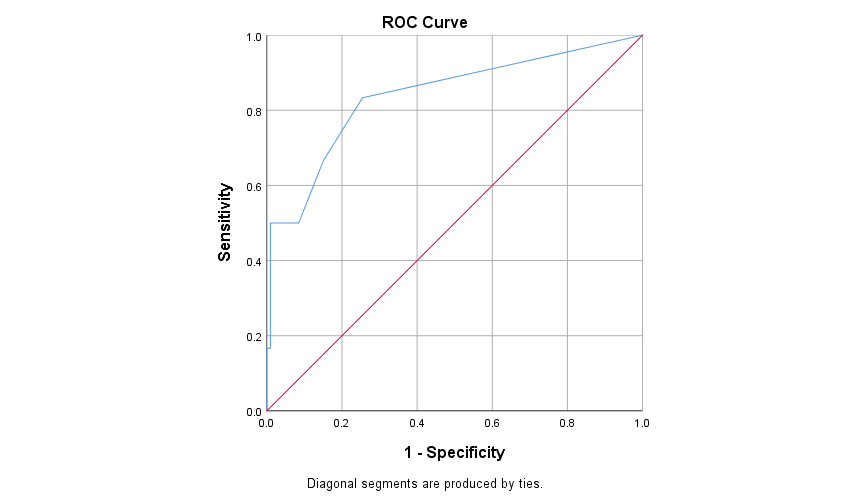

Supplement: Supplementary file 1 [file Datasheet1.docx]
